# The Stress Acceleration Hypothesis of Nightmares

**DOI:** 10.3389/fneur.2017.00201

**Published:** 2017-06-01

**Authors:** Tore Nielsen

**Affiliations:** ^1^Dream and Nightmare Laboratory, Center for Advanced Research in Sleep Medicine, CIUSSS-NÎM – Hôpital du Sacré-Coeur de Montréal, Montreal, QC, Canada; ^2^Department of Psychiatry, Université de Montreal, Montreal, QC, Canada

**Keywords:** nightmares, stress acceleration, affective disorders, infantile amnesia, adverse childhood experiences, trauma

## Abstract

Adverse childhood experiences can deleteriously affect future physical and mental health, increasing risk for many illnesses, including psychiatric problems, sleep disorders, and, according to the present hypothesis, idiopathic nightmares. Much like post-traumatic nightmares, which are triggered by trauma and lead to recurrent emotional dreaming about the trauma, idiopathic nightmares are hypothesized to originate in early adverse experiences that lead in later life to the expression of early memories and emotions in dream content. Accordingly, the objectives of this paper are to (1) review existing literature on sleep, dreaming and nightmares in relation to early adverse experiences, drawing upon both empirical studies of dreaming and nightmares and books and chapters by recognized nightmare experts and (2) propose a new approach to explaining nightmares that is based upon the Stress Acceleration Hypothesis of mental illness. The latter stipulates that susceptibility to mental illness is increased by adversity occurring during a developmentally sensitive window for emotional maturation—the *infantile amnesia period—*that ends around age 3½. Early adversity accelerates the neural and behavioral maturation of emotional systems governing the expression, learning, and extinction of fear memories and may afford short-term adaptive value. But it also engenders long-term dysfunctional consequences including an increased risk for nightmares. Two mechanisms are proposed: (1) disruption of infantile amnesia allows normally forgotten early childhood memories to influence later emotions, cognitions and behavior, including the common expression of threats in nightmares; (2) alterations of normal emotion regulation processes of both waking and sleep lead to increased fear sensitivity and less effective fear extinction. These changes influence an affect network previously hypothesized to regulate fear extinction during REM sleep, disruption of which leads to nightmares. This network consists of a fear circuit that includes amygdala, hippocampus, and medial prefrontal cortex and whose substantial overlap with the stress acceleration findings allows the latter to be incorporated into a wider, more developmentally coherent framework.

## Explaining Idiopathic Nightmares

This work proposes a novel explanation for idiopathic nightmares, i.e., nightmares for which the causes are still unknown. Such nightmares are common, affecting almost everyone at least occasionally but rising to the level of a clinical problem for about 5% of the population (1–5). Although idiopathic nightmares may be distinguished from nightmares for which a proximal cause is more obvious, e.g., trauma, drug ingestion, epilepsy, and bereavement (6), it is still unclear why only some individuals succumb to nightmares in these circumstances and where, ultimately, the unusual dysphoria of nightmares originates. Furthermore, there are many potential causes of idiopathic nightmares that have been treated in the literature, such as hereditary factors (7), psychological distress (8), an alexithymic personality style (9, 10), or schizoid personality style (11), even eating certain foods before bed [e.g., cheese and dairy; (12)], but none of these has been reliably shown to account for a majority of nightmares. Furthermore, some writers have speculated that nightmares are an active, functional phenomenon which has adaptive consequences for emotion regulation (13). A variety of mechanisms have been proposed, including desomatization (14), affective downregulation (15), and fear extinction (16). One evolutionary theory of nightmare function (17) proposes that nightmares reflect the operation of an endogenous mechanism that bestows adaptive advantage by simulating rehearsals of threat perception and avoidance. Some evidence supports the idea (18) but this and other functional theories are far from accepted as proven. In short, numerous potential causes of idiopathic nightmares have been proposed, even systematized into theories of nightmare function, but their validity remains contentious (13) and none has proven to adequately explain the bulk of nightmares.

Idiopathic nightmares are often so frequent or distressing that they warrant a psychiatric diagnosis, i.e., Nightmare Disorder (1, 2), and require targeted treatment. For some individuals, nightmares are chronic, having been present since early childhood. For others, they disappear after childhood only to resurface in adolescence or adulthood, often in response to seemingly benign events such as changing schools, retiring, viewing a frightening movie or having a minor accident. Idiopathic nightmares are also frequently comorbid with psychiatric conditions such as social anxiety, phobia, or depression and may presage future PTSD or self-harm and suicidal behaviors. But in many cases, nightmares occur with frequencies and associated distress that are low and tolerable and not considered by individuals or health professionals to be pathological. In short, idiopathic nightmares span a wide spectrum of severity and predict other illnesses but are often considered benign.

So is there a cause for such nightmares? And could this cause account for both frequent, distressing and infrequent, benign types of nightmares? *The present work suggests that the answer to these questions is yes, and that their ultimate cause may be similar to that of a closely related phenomenon: the post-traumatic nightmare*. Both idiopathic and post-traumatic nightmares may be due to a vulnerability triggered by adverse experiences. What may be unique to idiopathic nightmares, however, is that the adverse experiences at fault occur early in childhood and are either forgotten or—because they are not considered traumatic by conventional standards—dismissed as causally irrelevant.

But the possible influence of early adversity on nightmare etiology should be re-examined in light of a growing awareness of the high prevalence and multiplicity of adversities in childhood and of the need to broaden and assess the relative severity of these adversities at different critical ages (19–21). To illustrate the magnitude of the situation, one US population study spanning 2004–2011 of over 5.5 million maltreated children (ages 1–18) found a cumulative prevalence of state-confirmed maltreatment in 2011 of 12.5%; fully 46% of these children were maltreated before their fifth birthday (22). Many other forms of severe adversity are not typically included in such maltreatment reports: bullying, discrimination, witnessing violence, media/technology exposures, and foster care placement, to name only a few (19). Consideration of such adverse experiences vastly widens the scope of possible causes for idiopathic nightmares and links the latter conceptually to the etiology of post-traumatic nightmares.

The present investigation explores this central question of nightmares’ origins in early adversity. Its objectives are twofold: (1) to review the literature relating dreaming, nightmares and sleep more generally to early adverse experiences, drawing upon both empirical studies and books and book chapters by recognized nightmare experts and (2) to propose a new approach to explaining nightmares that is based upon the Stress Acceleration Hypothesis of mental illness.

### A Spectrum of Nightmare Severity

The present approach considers idiopathic and post-traumatic nightmares to be phenomena related on a nightmare severity spectrum (5). This implies that idiopathic nightmares may constitute a *subthreshold, preclinical*, or *partial* form of PTSD (23). The two types of nightmares are often distinguished on the grounds that post-traumatic nightmares reproduce features of the trauma—so-called *replicative nightmares* reported by 42–53% of PTSD patients (24–26)—or that they represent a trauma in symbolic or distorted form (24, 27). In fact, emotions are the element that is most likely to influence PTSD dreams; up to 80% of dreams replay trauma-related emotions, irrespective of whether these replicate other trauma features (28). Idiopathic nightmares, while not replicating prior trauma *per se*, are similar in that they produce recurrent, dysphoric themes (29) that relate to waking stress and emotional concerns (30). In light of such parallels, distinctions between replicative and idiopathic nightmares need reevaluation. This would require, as a first step, identifying participants’ early adverse experiences and, subsequently, determining if memories of these experiences—emotions in particular—appear in nightmares. This type of comparative evaluation has not been accomplished. Indeed, it is rare to see any comparison between post-traumatic and idiopathic nightmares [but see Ref. (31)].

Thus, whereas causal links between trauma and post-traumatic nightmares are well established, similar links between less severe adversities and idiopathic nightmares are only beginning to come into clearer focus. The unification of post-traumatic and idiopathic nightmares along a single adversity-related spectrum may provide answers to the long-standing puzzle of the origins of nightmares and may also provide a unified framework for answering questions about the variable appearance of nightmares over the lifespan, about the frequent comorbidities of nightmares and about nightmares constituting a risk factor for other illnesses. The proposed framework for explaining idiopathic nightmares points to the necessity of evaluating forms of early adversity and to the further possibility that memories for such adversity—emotional memories especially—resurface in idiopathic nightmares.

## The Ravages of Early Adversity

Altricial mammals, such as humans, must care for their young through a long, vulnerable period of infancy. And although the threats of infancy are largely buffered by parental presence (32) and the rapid forgetting of fearful events ensured by *infantile amnesia* for this developmental period (33), infants are frequently exposed to many forms of adversity. Such exposure is a potent risk factor for physical and mental health problems in later life [review in Ref. (34)]. Early adversity increases risk for diverse physical conditions: obesity/diabetes, asthma, lung cancer, autoimmune conditions, migraine, cardiovascular disease, and health-risk behaviors (35, 36); reviews in Ref. (37–39). In fact, adversity increases mortality risk in a graded fashion. Baboons exposed to three or more sources of early adversity die a median of 10 years earlier than do those exposed to one or no sources (40). Similarly, adult humans exposed to six or more childhood adverse experiences die on average 20 years earlier than do adults with no exposure (41).

Early adversity also leads to psychiatric disorders such as psychosis, PTSD, borderline personality, anxiety, depression, and suicide [reviews in Ref. (38, 42–44)]. One study examining 20 DSM-IV anxiety, mood, substance, and disruptive behavior disorders found early adversity associated with 45% of childhood onset disorders and 26–32% of later onset disorders (44). These relationships are also graded; more severe or sustained exposures produce greater health consequences (45, 46).

Sleep disorders are among the many illnesses linked to early adversity [reviews in Ref. (47, 48)]. Relationships have been documented for poor sleep quality, feeling unrested (49), narcolepsy, sleep apnea (50), sleep violence (51), disruptive nocturnal behaviors (52), trouble falling/staying asleep (47, 53), and sleep terrors (54) among others. Such studies, too, find graded relationships with symptom severity (48, 53), e.g., each additional childhood adversity is associated with a 10% increased risk of troubled sleep in adulthood (47).

### Nightmares Are Associated with Early Adversity

Nightmares are central among the sleep disorders associated with early adversity and the relevant literature is summarized below. However, because available studies vary in the precision with which the age of early adversity is assessed, support for the role of early adversity as stipulated by the Stress Acceleration Hypothesis is problematic. Studies that probe generically about “past adversity” or “childhood adversity” provide age estimates that cannot be pinpointed as the present approach requires (see Stress-Induced Foreshortening of the Infantile Amnesia Period). Nonetheless, findings from several studies converge in supporting the general notion that early adversity increases nightmare risk.

The studies least carefully controlled for age of adversity—all cross-sectional or retrospective in nature—evaluated childhood adversities without specifying a specific age range. One assessment of 30 lifelong nightmare sufferers (55) found that a “major life event” preceding nightmare onset could be identified by 60% of participants. A second study (50) showed that the likelihood of participants reporting a traumatic event “in childhood” was higher among those currently reporting nightmares *often* (55% or 11/20) than only *sometimes* (27% or 46/170) or *never* (24% or 24/100) combined (*X*_2_ = 7.81, *p* = 0.005). This relationship was strong (*p* = 0.008) for self-reported physical abuse, for which 35, 11, and 9% of the *often, sometimes*, and *never* groups, respectively, reported past incidents, but was absent for maternal separation, maternal loss, and sexual abuse. Duval et al. (56) reported that 19- to 24-year-old women who reported abuse in childhood or adolescence (age unspecified) currently recalled more nightmares. This relationship was graded for *none, low, moderate*, and *high* abuse levels on measures of monthly bad dreams (no awakening; means: 2.18, 2.39, 2.85, 3.99) and nightmares (means: 0.90, 0.88, 0.99, 2.69). However, the Childhood Trauma Questionnaire used did not permit distinguishing childhood from adolescent abuse.

Other retrospective studies have differentiated childhood and adolescent adversities. One survey of 539 Canadian women (57) found that histories of sexual or physical abuse predicted frequent nightmares, frequent recurrent nightmare themes, and more difficulties returning to sleep after nightmares but no differences between participants abused before or after age 14. A second study (58) demonstrated that adverse experiences before age 14, compared to no adversity, led to more frequent recall of nightmares, more distress from nightmares, and greater waking impact of nightmares. A third study (59) showed that 7- to 19-year-old Kurdish children (*N* = 122) with high levels of trauma reported more nightmare awakenings, more dreams with death, destruction, fear, anger, hostility, and anxiety and fewer dreams with bizarreness/symbolism, narrative quality, and a pleasant atmosphere than did children with low levels of trauma.

The studies most precisely specifying age have evaluated adversity in the infantile years. One longitudinal study of 6,050 UK children (60) demonstrated robust links between family adversity occurring from pregnancy to age 4 and later nightmares at ages 2.5–6.8 years. It also revealed a link between early child temperament and persistent nightmares, a finding that replicates a longitudinal study of 987 Quebec children (61) in which mother ratings of the child’s difficult temperament at 5 months and anxiety at 17 months predicted bad dreams at 29 months—bad dreams which then largely persisted until 6 years of age.

A study of very early adversity (62) in a nationally representative sample of 5,020 Hungarian adults demonstrated an association between retrospectively reported maternal separation for at least a month in the first year of life and currently recalling frequent nightmares. The effect was independent of age, gender, and education, but not depression. However, a similar relationship between maternal separation and frequent dysphoric emotions, i.e., oppressive and unpleasant dreams, was also found and was independent of all confounding measures.

One retrospective study (63) provides new details about the nature and age of early adverse events in relation to nightmares using responses to the Traumatic Antecedents Questionnaire [TAQ; (64)] for 62 nightmare sufferers (42 females; *M*_age_: 24.3 ± 4.2 years; 2+ nightmares/week), and 61 matched controls (41 females; *M*_age_: 24.0 ± 4.6 years; <1 nightmare/month). The TAQ assesses eight forms of traumatic and non-traumatic adversity (e.g., *neglect, separation, emotional*, and *physical*) separately for four age strata (*years 0–6, 7–12, 13–18, adult*). Nightmare participants scored higher than controls for adversity at all ages; even for 0–6 years they reported higher *total adversity* (*p* = 0.005), *sexual* (*p* = 0.026), *emotional* (*p* = 0.011), *alcohol/drug* (*p* = 0.052), *witnessing* (*p* = 0.07), *physical* (*p* = 0.08), and *other* (*p* = 0.06) abuse scores than did controls. In short, nightmare participants reported more past adversity—both traumatic and non-traumatic—including during early childhood.

Item-by-item group comparisons for the 0–6 age stratum in this study reinforced the notion that different types and degrees of early adversity befell the nightmare group (Table 1). Item scores that were elevated for the nightmare group included some that were clearly traumatic, e.g., *#29: I was in a situation in which I was convinced that I would be physically injured or lose my life*, and *#31*: *I saw dead bodies*, and possibly *#34: I saw sexual things that scared me*, but also items that were general in nature (*# 9: My parents confided things in me that made me feel uncomfortable; #18: The rules in my family were unclear and inconsistent*), not clearly recalled (*#40: Something terrible happened to me that still remains a mystery to me*), or even seemingly benign (*#3: not excelling at something*). This diversity and, especially, evidence that very general adverse events were reported by nightmare participants, reinforces the observation that many participants could not name specific events that might have triggered their nightmares, even though they believed such events had occurred. Specifically, even though 53.2% (33/62) of the nightmare group claimed an event preceded onset of their nightmares, only 35.5% (22/62) could describe one. For the control group, 59.0% (36/61) claimed an event preceding their nightmares and only 24.6 (15/61) described one. For both groups, a variety of possible trigger events was listed, but by far the most common were interpersonal events, usually involving the parents: death of a parent or other family member, parental divorce or separation, family discord, and bullying; only rarely was a clearly traumatic event described, i.e., a sexual assault in one nightmare participant.

**Table 1:** **Traumatic Antecedents Questionnaire (TAQ) items for 0–6 years of age that discriminate between Nightmare and Control groups**.

TAQ item (0–6 years of age)	Nightmare			Control				
	
	Mean	#*P*	%*P*	Mean	#*P*	%*P*	MWU^a^	*p*^a^
3. I was really good at something (like sports, a hobby, school, work, or some creative activity)	2.28	52/58	89.7	2.66	52/53	98.1	−1.75	0.081
9. My parents confided things in me that made me feel uncomfortable	0.55	16/56	28.6	0.19	6/53	11.3	2.29	0.022*
18. The rules in my family were unclear and inconsistent	0.71	19/59	32.2	0.26	11/58	19.0	2.00	0.045*
25. Someone in my family had a problem with alcohol and/or drugs	0.60	14/58	24.1	0.28	8/61	13.1	1.83	0.071
29. I was in a situation in which I was convinced that I would be physically injured or lose my life	0.10	3/60	5.0	0.00	0/61	0.0	1.76	0.078
31. I saw dead bodies	0.08	4/61	6.6	0.00	0/61	0.0	2.03	0.043*
34. I saw sexual things that scared me	0.20	4/61	6.6	0.00	0/61	0.0	2.54	0.011*
38. I believe that one of my brothers or sisters was sexually molested	0.16	4/58	6.9	0.00	0/58	0.0	2.04	0.041*
39. I have had another very frightening or traumatic experience where I felt intense fear, helpless, or horrified	0.33	4/58	6.9	0.02	1/60	1.7	2.92	0.004**
40. Something terrible happened to me that still remains a mystery to me	0.35	10/58	17.2	0.02	1/60	1.7	2.96	0.003**

*Mean, mean TAQ item rating; #P, number of participants; %P, percent of participants*.

^a^Mann–Whitney *U* standardized test statistic, two-tailed; *p < 0.05; **p < 0.01. From Ref. (63).

Finally, it is noteworthy that, as with other physical and psychiatric illnesses, relationships between adversity and nightmares appears graded, e.g., duration of childhood sexual abuse correlates positively with adult nightmare frequency and distress (65).

To summarize, research converges on the notion that risk for developing nightmares increases following early adversity exposure. While studies vary in the precision with which they have determined a participants’ age when adversity occurred, some suggest that events in the infantile amnesia period—even as early as the first year of life—influence adult nightmares. Some also suggest that adverse events need not be blatantly traumatic to have an effect; different types and severities of adversity are reported. Nightmare sufferers themselves describe experiences that range from seemingly benign (*birth of a sibling*) to generally disagreeable (*inconsistent parental rules*) to serious but non-traumatic (*separation of parents*) to outright traumatic (*seeing dead bodies*). Moreover, although many suspect past adverse events, they cannot specify them (*something terrible happened to me that still remains a mystery to me*). In short, nightmares may be influenced by experiences that are not obviously adverse, that may not be appreciated as causal, or that may not be clearly remembered, but that have a lingering emotional impact. If so, the causes of idiopathic nightmares may be of the same general ilk as those for post-traumatic nightmares only less severe and, possibly, cumulative in nature. This principle is nicely illustrated in a careful clinical study conducted by Hartmann (11, 66, 67) described next.

### Hartmann’s Clinical Evaluation of Nightmare Sufferers

Hartmann’s (11, 66, 67) work highlights the sometimes subtle nature of early adversity. He assessed 50 individuals with lifelong nightmares (3–4 nightmares/week) for evidence of a past history of trauma. He questioned them about childhood experiences in an attempt to “… *find any unusual characteristics, including the possibility of any traumatic events or unusual family relationships or dynamics that might have led to the onset of nightmares*.” (p. 67). He was confident in his participants’ abilities to accurately report these experiences, finding that they remembered their early childhoods “unusually well” (p. 67). Although Hartmann did not find clear evidence of early trauma, his participants did report being quite sensitive as young children and possibly more vulnerable to milder forms of adversity. For example, he observed that many of his participants had a younger sibling born when they were 1–3 years of age:

… they did appear to have taken the birth of the sibling unusually hard … It seemed rather that the psychological makeup of the nightmare sufferers was perhaps already somewhat unusual, even at the age of one to three, so that the birth of a sibling was especially painful or traumatic to them. (p. 70).

He concluded that a traumatic etiology could not be completely ruled out and that “… *a relatively ordinary event, such as the birth of a younger sibling, could have been felt to be especially painful and traumatic; most of them may indeed have had a series of “traumas” of this kind during childhood*.” (pp. 106–107).

Thus, according to this nightmare expert, a combination of “sensitivity” and events as seemingly benign as birth of a sibling might be sufficient to incite nightmares. The possibility was considered by researchers (68, 69) who assessed birth order in relation to nightmare frequency under the assumption that firstborn children would suffer adversity in the form of “dethronement” by the birth of a sibling. Their findings supported the assumption: the frequency of frightening dreams among firstborn children (15.2%) was over twice that of last-born children (6.7%; *p* < 0.02)—independent of confounders age, sex, and sibship size. They replicated the findings as well [but cf. Ref. (70)]. Birth of a sibling might seem to be an innocuous event in a child’s life, and birth order studies have even found firstborns to possess positive qualities such as high success and achievement [review in Ref. (71)]; however, several studies in the same review also link firstborns to emotionally maladaptive attributes such as “most fearful in new situations” (five studies), “most vulnerable to stress” (three studies), and “least emotionality” (three studies).

The following illustrates a preschool child’s intense reaction to the presumably adverse event of a sibling’s birth: “… *Harry was three and a half years old when his youngest brother was born*…*Just before his brother’s birth he became aggressive, began hitting his brothers and sisters, and became angry at his mother and her large stomach and told her he was going to open up her stomach and cut up her new baby. About a month after the baby’s birth, Harry began to have nightmares from which he awoke in terror. He dreamt that someone was coming to hurt him, that monsters were after him, that a large woman was coming to kill him. Here the nightmares clearly seem to be portraying a very specific fear of punishment for his aggressive wishes*.” [(72), pp. 65–66].

There are other signs in the Hartmann histories of possible early adversity. Significantly more of his nightmare group had close relatives with a psychiatric condition, interactions with whom could have been a source of adversity. Further, 18/50 (36%) of his patients fit a DSM diagnosis of schizophrenia, schizotypal disorder, or borderline personality disorder; many others showed preclinical signs; there were no such signs among controls. Similar clinical findings were reported by Kales et al. (55) who noted higher levels of schizotypy and emotional problems (e.g., quick to withdraw emotionally, overreact to perceived mistreatment and considered committing suicide) among nightmare participants than among controls. Furthermore, more nightmare than control participants reported emotional problems in their families and that family members had committed suicide.

### Nightmares Are Developmentally Persistent

If there is growing evidence of emotion-mediated causal links between early adversity and later idiopathic nightmares, much less is known about when nightmares may arise in relation to this adversity. Some evidence suggests that nightmares often arise in childhood and persist until later in life. There may be a peak in nightmare prevalence early in adolescence [e.g., Ref. (73)], but another study of >950 children found that bad dreams were present in some as young as 29 months of age (61). In fact, for bad dreams rated by mothers as occurring *often* or *always* the prevalences for children 29, 41, 50, 60, and 72 months of age were 2.4, 3.9, 2.2, 2.6, and 1.5%, respectively. Thus, there was a peak prevalence at 41 months (3.5 years) compared with other early ages. This peak is particularly noteworthy considering that 3.5 years of age is the widely accepted infantile amnesia upper boundary (see later section). These analyses further showed that when bad dreams did occur in early childhood, they tended to persist over time; of the 68.2% of children who had bad dreams at 29 months, 82.0% still had them at 41 months, and, of these, 88.3% still had them at 50 months, 87.3% at 60 months, and 89.7% at 72 months (61). A similar longitudinal stability was found for 851 8- to 11-year-old children assessed at 3 time-points over 2 years (74).

Although nightmares may start early in life and persist as a lifelong problem, this is not always the case. The impact of early adversity on nightmares may surface only after some variable delay. For example, an accumulation of milder adverse experiences might increase one’s susceptibility to nightmares over time, with nightmares appearing only after an adversity threshold has been attained. Their appearance may thus be (imprecisely) blamed on seemingly minor events—the last adversity encountered. Similar considerations pertain to post-traumatic nightmares. Sometimes these emerge relatively promptly post-trauma but sometimes they appear only after a substantive delay, so-called *delayed-onset PTSD* (75). An accumulation of trauma experiences is known to increase risk for future PTSD (76, 77).

## The Stress Acceleration Hypothesis of Mental Illness

The present section explores application of the Stress Acceleration Hypothesis of mental illness [SAH-MI; (78)] specifically to the pathogenesis of nightmares. The SAH-MI in its simplest form stipulates that susceptibility to mental illness is increased by adverse experiences that occur during an early, developmentally sensitive window. This sensitive window exists in other altricial species, e.g., rats (33) and dogs (79), and corresponds in humans to the period from birth to approximately 3.5 years of age (80). It is commonly referred to as the *infantile amnesia* period (33, 81) and is defined more fully later. Early exposure to adversity is postulated to trigger a pattern of changes that is common across species, specifically, a precocious maturation of emotional regulation systems such that basic emotion processes, particularly those governing fear expression, learning and extinction for the infant, shift prematurely to an adult-like form. This acceleration may afford short-term adaptive advantages, such as better emotional self-regulation in the absence of adequate caregiving, but its long-term consequences may be disproportionately damaging; it may lead to anxiety, depression and an increased risk for mental illness—including, according to the present thesis, the manifestation of nightmares.

The SAH-MI is grounded in evolutionary theories of development and is supported by substantial behavioral and neurocognitive research with both human and non-human subjects (9, 78). It is consistent with other theories, such as psychosocial acceleration theory, and findings that support them, such as evidence that the timing of puberty onset in females is accelerated after socioemotional adversity in the first 5 years of life [review in Ref. (82)]. In line with these views, the present work considers stress acceleration as a mechanistic explanation for nightmares and, in particular, to explain how adversity may induce cognitive, behavioral, and neural changes to emotion regulation processes during both wakefulness and sleep, processes that are particularly germane to explaining nightmare production.

### Stress Acceleration Affects Emotional Regulation

A comprehensive set of literature reviews (9, 32, 78, 83–86) details the hypothesis that early environmental factors, e.g., early caregiving or the lack thereof, modulate the timing and course of emotional development. The principal tenets of this hypothesis are summarized in Table 2, sections A and B; additional assumptions as applied here to nightmares are summarized in section C. The previous authors’ works are predicated on several general assumptions about evolutionary pressures on development (assumption 1), the role of early adversity in future mental illness (assumption 2), and the preservation of emotional functioning across species (assumption 3). Readers are directed to the original reviews for further clarification of, and empirical support for, these general assumptions. Assumption 3, that emotion neurocircuitry and function is preserved across species (3, 4), is critical to both the SAH-MI and the current thesis in that it allows comparative analysis of findings from research based on both human and non-human subjects.

**Table 2:** **Assumptions of the Stress Acceleration Hypothesis of mental illness (A, B) and additional assumptions proposed for nightmares (C)**.

(A) General assumptions
Evolutionary: developmental trajectories respond to the environment to confer survival advantage^a^ Developmental: early adverse experiences influence later mental illness^b^: Focus on adverse caregiving: abuse, neglect, maternal separation Also relevant: bullying, witnessing violence, death, trauma, etc. Neural: neurocircuitry of emotional functioning is highly preserved across species^c^: Infantile amnesia period (in altricial mammals) Affect network: limbic-prefrontal connections

(B) Assumptions about stress-induced acceleration 4. Adversity accelerates the nature and timing of early emotional behavior Foreshortening of infantile amnesia period^d^ o persisting infantile influences on adult behavior Fast-tracking of adult-like emotional behavior^e^ o heightened fear expression o relapse-prone fear extinction 5. Adversity accelerates the nature and timing of emotion neurobiology^f^ Affect network: amygdala, medial prefrontal cortex, hippocampus, anterior cingulate cortex

(C) Assumptions pertinent to nightmares 6. Accelerated early development of emotion circuitry sets the stage for nightmares Development of sleep-related emotion regulation processes (fear learning, extinction) parallels that of waking-related processes o qualitative change in processes after infantile amnesia boundary o adversity-induced acceleration of processes Foreshortening of infantile amnesia period increases access to normally forgotten memory traces during dreaming: o primordial feelings o infantile memories Acceleration of sleep-related emotion regulation mechanisms o NREM sleep mechanisms (sleep spindles) o REM sleep mechanisms (%REM, theta EEG) 7. Adversity-induced acceleration produces alterations common to nightmares, comorbidities, and correlates Nightmares are comorbid with anxiety, depression, schizophrenia spectrum, suicide/self-harm, PTSD Nightmares may be a preclinical (partial) form of PTSD Nightmare sufferers may be high in environmental sensitivity, creativity, recall of early memories

*^a^(87, 88)*; *^b^(44, 89)*; *^c^(33, 90)*; *^d^(91)*; *^e^(78)*; *^f^(92)*.

### Stress Acceleration Has Cognitive, Behavioral, and Neural Effects

As shown in Table 2, sections B4 and B5, the SAH-MI claims that early adversity accelerates development of both emotional behavior and the behavior’s neurobiological underpinnings. Literature supporting these claims is extensive, especially for rodent models of emotion (9, 78), and only some representative findings are discussed here. In relating this literature to the pathogenesis of nightmares, it will be useful to distinguish among effects that are more specifically pertinent to human cognitive–emotional processes, including effects on emotional imagery, emotional memory, and sleep. These distinctions will highlight processes central to nightmare production and phenomenology but that are not typically considered in relation to SAH effects of emotional functioning more generally.

## The Stress Acceleration Hypothesis of Nightmares

**Figure 1:**
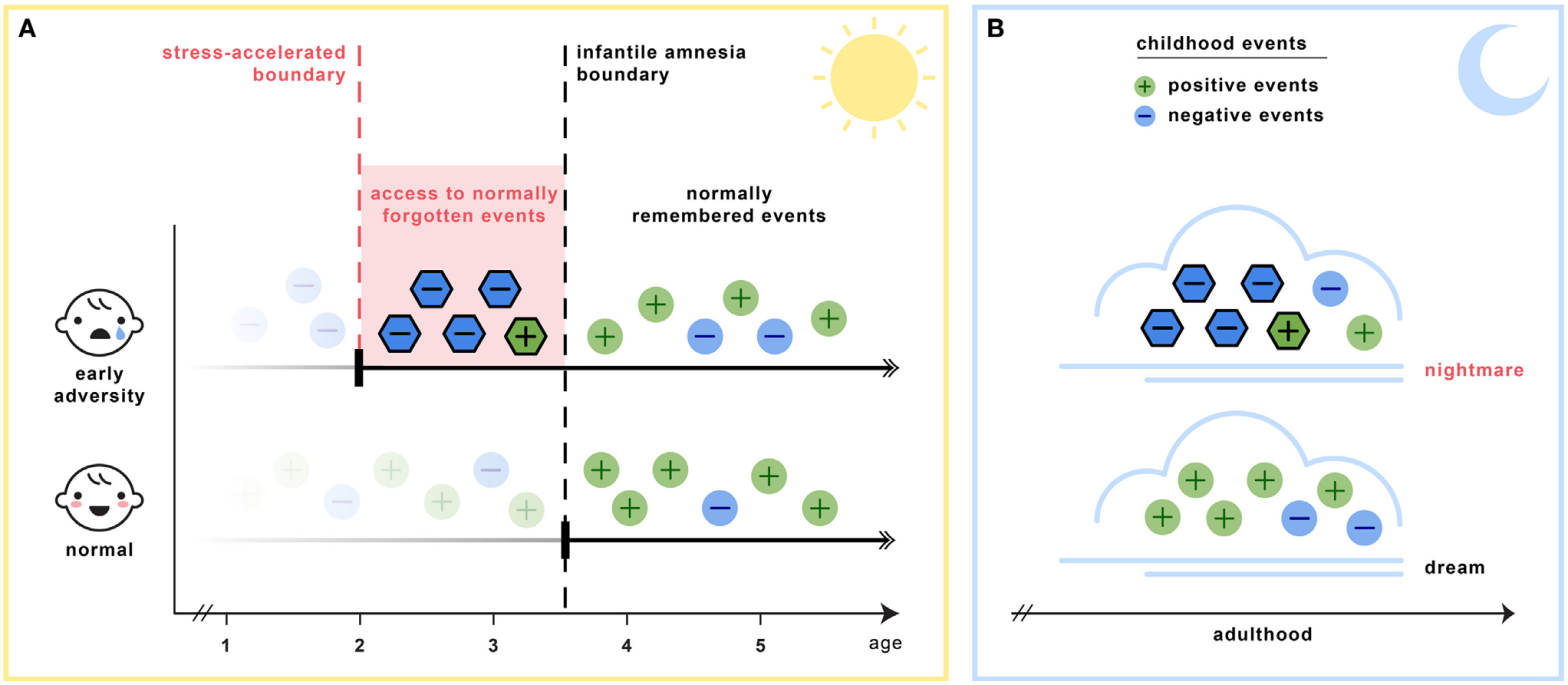
**The stress acceleration hypothesis of nightmares stipulates that (A) intensely emotional events originating in the infantile amnesia period are normally inhibited to adult recall at the infantile amnesia boundary, which ends at about 3.5 years of age (vertical black line)**. But early adverse experiences may cause this boundary to be advanced (vertical red line), rendering these early events more accessible to memory (red region). **(B)** Because dream formation draws upon early memories, the dream experiences of adults who had suffered early adversity may be nightmarish because they include some of these normally forgotten elements (nightmare cloud). Dreams of adults with no early adversity will not include these early memory elements (dream cloud). (Image credit: Sabrina Nielsen).

Section C6 of Table 2 details the key assumptions of the SAH-MI as it applies to nightmares (see also Figure 1). First, there occurs a foreshortening of the infantile amnesia period—with parallel changes hypothesized to occur for dreaming such that, in later development, access to both primordial feelings and infantile memories is increased. Second, there occurs a precocious maturation of emotional memory processes which both sustains feelings and memories from the infantile period and alters emotional reactions to ongoing events. Third, there is modification of REM and NREM mechanisms implicated in emotion memory and regulation. Thus, early adversity not only displaces an evolutionarily preserved amnestic barrier to early memories, but it alters emotional learning in such a way that these early memories—including memories for the adversity itself—are given a much longer lifespan and a greater influence over adolescent and adult development. These influences on emotional development will be described in turn.

### Stress-Induced Foreshortening of the Infantile Amnesia Period

The nightmare application of the SAH hypothesis stipulates, in accordance with other theories, that adversity induces a foreshortening of the infantile amnesia period and that this change renders the individual vulnerable to the influences of normally forgotten early emotions and memories. However, the present theory goes further to stipulate that this change will have effects on emotion and memory processes in both awake and sleep states. As the hypothesized source of nightmares, infantile amnesia foreshortening should be particularly likely to affect neural processes underlying dreaming and sleep-related emotional memory consolidation. Evidence pertaining to the presumed effects of infantile amnesia foreshortening on emotions and memory during both waking and dreaming is considered in the next sections.

#### Infantile Amnesia: Waking State

The phenomenon of infantile amnesia, or the absence of memory for events from the first few years of life, was first described by Henri and Henri (93) and later named and elaborated by Freud (81, 94). Freud considered infantile amnesia a paradoxical riddle: on the one hand, most early memories are forgotten despite the vivid nature of early experiences (“… *no trace is found in an adult’s memory of impressions dating from that time which are important, impressive and rich in affect*.” 1901, p. 83) and, on the other hand, such early experiences profoundly influence adult behavior (“…*the very impressions which we have forgotten have nevertheless left the deepest traces in our psychic life, and acted as determinants for our whole future development*.” 1905, p. 28). To illustrate the impact of this thinking, Breuer and Freud (95) proposed that “hysteria,” an early conceptualization of anxiety disorder, was due to a fearful event remaining in memory and activating anxious thoughts and maladaptive behaviors. This notion that early memories contribute to anxiety disorders is still widely held [review in Ref. (96)] and is a component of the present explanatory framework for nightmares.

Although Freud’s notion that both dreams and neurotic symptoms express unconscious early impressions (“infantile wishes”) was a cornerstone psychoanalytic concept, it was also a constant challenge to him forming a coherent explanation of nightmares: nightmares do not on the surface appear to fulfill any kind of wish as commonly understood. Yet, the notion of enduring unconscious influence is surprisingly consistent with stress acceleration frameworks and with the current suggestion that early adverse impressions influence nightmares.^1^

**Figure 2:**
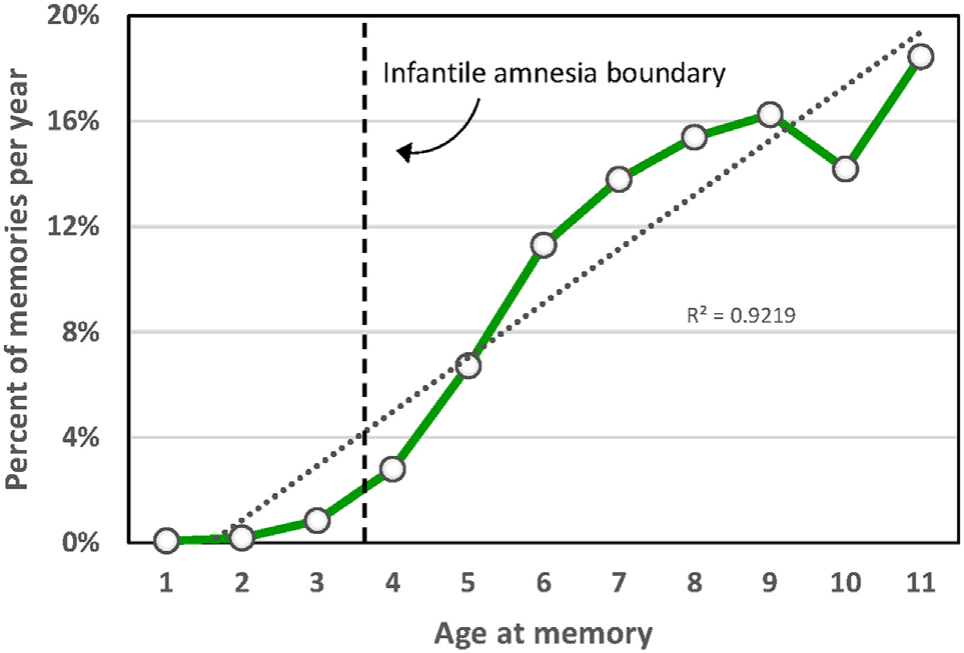
**Distribution of participant ages at the time of childhood autobiographical memories aggregated over 11,289 memories from 762 participants in 33 studies [from Ref. (80)]**.

The paradoxical nature of infantile amnesia pointed out by Freud influenced developmental theorists [e.g., Ref. (98, 99)] and was supported empirically in the early twentieth century [reviews in Ref. (100, 101)] and more recently [reviews in Ref. (33, 91)]. A key development was the demonstration that infantile amnesia is not restricted to human infants; rather, it is a biologically determined developmental milestone demonstrated in rats (102) and other altricial species (103, 104). Campbell and Campbell first demonstrated its cross-species generality: both infant and adult rats showed equivalent levels of learning and expressing of fear when tested immediately after training, but infants alone showed a rapid rate of forgetting when tested at later times. This directly parallels research showing that both 3- and 4-year-old children form episodic memories, but that 3-year olds fail to retain those memories following delays as brief as 30 min, whereas 4-year olds retain them up to a week later (105). Studies of adults’ retrospective dating of autobiographical memories demonstrate that few memories predate age 4; Rubin’s (80) assessment of over 11,000 such memories reported for age 10 and younger found that only 3.9% occurred before age 4, and the percentage rose sharply afterward (Figure 2). Such findings, together with a large neurobiological literature [review in Ref. (33)], indicate that infantile amnesia is a normal developmental feature of altricial species and, in humans, sets in at about 3.5 years of age. The estimated boundary of 3.5 years might even be an underestimate. If early adversity indeed foreshortens this boundary as the SAH-MI stipulates and if some of Rubin’s (80) participants had experienced prior adversity as might be expected from epidemiological studies (106, 107), then the estimated normal boundary of 3.5 years may be later, possibly even closer to 4 years. More detailed study of this point is needed.

Although early adversity may advance the infantile amnesia boundary, memory for adversity may be affected in different ways for children of different ages. Terr (108) found that the age range of 2.5–3 years was critical; children younger than 28 months when traumatized had only the spottiest of recall of the trauma, whereas older children had more complete recall. Field studies of injured young children confirm such observations. Children aged 18 months to 5 years who underwent emergency room visits for burns, lacerations, fractures, etc. and were interviewed within a few days and again 6 months later recalled very little at either time point when they were younger than 30 months at the time of the injury, but recalled very well when they were older than this (109). Similarly, children aged 12–33 months who had experienced injuries and emergency room treatments could not report their experiences either immediately or 2 years later when they were younger than 18 months at the time of the injury, but older children could give more detailed reports at both times (110).

Accordingly, empirical determinations of infantile amnesia in the case of early trauma has produced a range of estimates, in part due to variations in the specificity and pertinence of probe questions but also because of factors such as culture, trauma distinctiveness, time elapsed since trauma, and reminders [review in Ref. (111)]. However, there is a general consensus that, because of limited language capacities, traumatic events experienced before the age of around 2 years are not accessible in verbal form but may manifest in behavioral or physiological ways, and that only “bits and pieces” of memories can be recalled verbally for trauma occurring up to 2.5 years of age (111). Thus, while early adversity may influence children as early as birth, differences in language competency may determine in what form—verbal vs. non-verbal—adversity-induced accelerations of the infantile amnesia boundary will be expressed.

#### Infantile Amnesia: Dreaming Parallels

As there is little research on dreaming in the infantile amnesia period, there is consequently little agreement about dreaming prevalence and characteristics at this time. There is no research whatsoever about whether a shift in dream recall or dream content at the end of the infantile amnesia period may parallel the transition described earlier for emotional memory. Available studies on children’s dreams are specifically weak on these points because they have not assessed children’s dreaming at the critical juncture of the infantile amnesia boundary, i.e., ages 2–3 years vs. ages 4 and older; rather, such studies either exclude very young children altogether or group them such that their ages straddle both sides of the boundary, e.g., 3–6 years. Some studies investigating early developmental changes in sleep neurophysiology do provide evidence relevant to the question; these are reviewed in a later section (see Evidence from Changes in Sleep Architecture). To date, the best available evidence about a potential dreaming parallel to infantile amnesia is to be found in a series of studies (112, 113) comparing a cohort of children aged 3–5 years of age with older cohorts (5–7, 7–9, 9–11, 11–13, and 13–15 years). The youngest cohort is still imperfect in that it straddles the infantile amnesia boundary, but reports of “dreaming” in this group nonetheless offer some compelling clues. Briefly, this work suggests that dreaming is impoverished or absent altogether during the infantile amnesia period and begins a shift to adult-like dreaming only as children cross the infantile amnesia boundary. Comparisons of these normative findings with observations on nightmares reported by traumatized children will allow us to address the more specific question of whether the phenomenon of infantile amnesia acceleration induces a parallel acceleration in dream production.

Foulkes’ studies (112–114) employed both home and polysomnographic dream collection methods and a longitudinal design that compared children with themselves multiple times over a 5-year span. He considered his “single most amazing finding” to be the puniness of dreaming among the 3- to 5-year-old cohort relative to that for older children and adults [(114), p. 56]. The youngsters’ recall of dreaming from any stage of sleep was rare, occurring on a median of 15% of REM sleep awakenings and 0% of non-REM sleep awakenings. And when some recall did occur, reports were exceedingly brief (median: 13 words/report for boys; 14 for girls) and depicted isolated events that were static (non-kinematic) and lacking narrative structure. The images were largely devoid of social interactions, either pleasant (4% of reports with content) or unpleasant (6%), and devoid of emotions (8%)—fear or anger was never seen. There were few self-movements (13%) or other types of motor activity (26%). Animals were the predominant characters in both girls’ (45%) and boys’ (33%) reports and these eclipsed family members (17%). These features, except for the predominance of animal characters, were all distinct from the dreams of older subjects. Foulkes (113) concluded that “… *these findings are quite unique, not merely against the standard of adult dreams, but also against that of children’s dreams at all other ages at which we studied them, including these very same children’s dreams two years later at ages 5-7*” (p. 48).

Perhaps even more striking in these results is the finding that, whereas the frequency of recalling dreams among all older children (from 5 to 15 years) was consistently correlated with measures of cognitive skills, especially with the visuospatial Block Design task, 3- to 5-year olds were unique in showing *no* such correlations. In fact, their dream reporting was associated with dependence on adults, talkativeness and verbal spontaneity, attributes Foulkes thought may have encouraged confabulation or dream report false positives. In other words, the paltry amount of dreaming that did occur among 3- to 5-year olds was relatively devoid of socioemotional imagery, unrelated to adult-like cognitive competency, and possibly confabulated, whereas the older children’s dreams did contain socioemotional imagery, did reflect their visuospatial competency, and was not likely confabulated. This pattern of results may constitute a nocturnal parallel to the normal developmental sequence of changes in emotional memory that leads up to the infantile amnesia boundary, specifically, a shift from rudimentary dreaming that is sparse, easily forgotten, and lacking mechanisms to represent emotions or social interactions, to an adult-like form that is more abundant, kinematic, and socioemotionally rich. These, and other, findings led Foulkes to conclude that a capacity for dreaming parallels waking state competency for visuospatial cognition and, since such skills are largely absent in 3- to 5-year olds, dreaming too is largely absent.

Foulkes’ results thus support the notion of a parallel between waking and sleeping development very early in life and, in particular, between a shift from infantile to adult-like emotional memory during wake and a shift from impoverished to socially rich adult-like dreaming during sleep. Accordingly, it is reasonable to expect that early adversity will induce an abnormal acceleration of this shift in dreaming processes just as it is does acceleration of fear memory processes during wakefulness. While research is also very limited on this point, there is some evidence that early adversity is associated with children having nightmares before they reach the age of the infantile amnesia boundary. This evidence is largely clinical and anecdotal [review in Ref. (115)], although some new empirical findings about remembering very early dreams are presented in a later section.

Terr (115), Mack (116), and Hartmann (67) discuss accounts of apparent nightmares by children as young as 8–13 months, but these are clearly exceptional cases. The authors interpret context, behavioral indicators (“dream-enactments”) and rudimentary verbalizations to infer that such experiences are nightmares. Hartmann (67) suggests that a large number of the “waking up crying” episodes of children around age 1 are preverbal nightmares but acknowledges that they may be sleep terrors, which are at their peak prevalence (34%) around this age (117). Terr (118), as well, suggests that “*infants physically demonstrate that they are dreaming by making mouthing movements or little sounds in their sleep. Toddlers may scream from sleep without awakening, but this kind of dreaming is often too primitive and inexpressive to establish that traumatic dreams are actually taking place*” (118). Terr also considers that these are sleep terror dreams^2^ and provides the example of a 3-year-old girl whose sister had been kidnapped from her school bus and buried alive: the girl reported her first ever dream to her mother: “‘*In a hole. I—in a hole,’ Marjorie cried out in the middle of the night. The dream, one of being buried alive, had been transmitted from the older sister, who had experienced premature burial and dreamed it several times, to her toddler sibling*.” (p. 241).

There is somewhat stronger evidence that adversity-induced nightmares can be recalled by children older than 2.5 yr. First, in one study (61) of 987 preschoolers aged 7 months to 6 years, parent ratings of bad dreams at a frequency of *often* or *always* in the months prior to testing occurred in 2.4% of children aged 29 months and 3.9% aged 41 months. Further, these measures were predicted by proxy measures of earlier adversity, such as mother ratings of the child at 5 months being difficult, restless or likely to cry, or at 17 months being anxious and emotionally disturbed. Second, in a clinical study (108) of 20 children with documented trauma that had occurred prior to age 5 (range: 6–58 months), 4 of the children (20%) were observed to remember trauma-related dreams—all of these children were older than 34 months when remembering the dreams. Another four children between 20 and 34 months screamed at night without parents being able to verify dream content.

#### Infantile Amnesia: A Wider View

Together, the preceding findings suggest a time line for the development of emotional memory processes during both waking and sleep states. The normal developmental sequence may be that children younger than age 3.5 learn, but fail to retain, emotional memories while awake but also dream very little and without socioemotional themes and feelings when asleep; they subsequently develop more adult-like capacities for both memory and dreaming after this age. Adverse experiences may accelerate this normal developmental sequence for both waking and sleeping processes such that intensely emotional memories and dreams are retained and recalled from abnormally early ages.

Children younger than 2.5 years have been observed to express reactions to traumatic experiences in non-verbal (or implicit) ways during both wakefulness and sleep. During wake, this is most commonly seen in the form of bodily responses, trauma-specific fears, reenactive behaviors, or repetitions of trauma-related movements during play (108). During sleep, they rarely recall nightmares but show behavioral signs of sleep disturbance that belie the adversity’s influence, including dream-enactments, vocalizations, and other sleep terror signs. Adversity may thus influence non-verbal and verbal expressions of emotional memory and dreaming differently for different ages. Prior to age 2.5, adversity may lead to changes in behavioral (implicit) memory systems and possibly also to an increased vulnerability for nightmares indexed only by behavioral markers (behavioral repetitions, sleep motricity) but which only manifest as nightmares *per se* later, as cognitive–emotional processes fully mature. Other factors, such as the severity or chronicity of an adversity or a child’s natural resilience mechanisms, may also prevent immediate expression of nightmares even while contributing to the founding of a nightmare vulnerability.

### Infantile Amnesia Foreshortening and Nightmares: The Evidence

Foreshortening of the infantile amnesia period may have weighty cognitive and neurobehavioral consequences for the development of emotional memory systems during both waking and sleep; these may be separated for convenience into influences on *primordial emotions* and *infantile memory elements* (Table 3). First, primordial emotions, which include feelings accompanying instinctive behaviors (121) and evolutionarily basic emotions (122), may leave traces that consciously or unconsciously influence waking cognitions, images, and behaviors. Judging from the poorly regulated emotional behaviors of young children (e.g., crying outbursts, temper tantrums, fits of joy, etc.), primordial emotions are likely to be more vivid, volatile, and uncontrolled than are emotions experienced by older children or adults. Primordial emotions include, for the present work, feelings of anxiety, fear, dread, and terror that constitute the predominant feelings in nightmares, but may also include other intense emotions; anger, sadness, disgust, joy, interest, and other basic emotions all develop early in ontogeny and are felt by children long before they develop language, symbolization or a self-concept (123, 124). This corresponds with the fact that as many as 30–51% of nightmares contain emotions other than fear, e.g., anger, sadness, or frustration (125).^3^

**Table 3:** **Acceleration factors combining to influence emotion functioning and nightmares**.

Factor	Implications for wake state	Implications for nightmares	Comorbidities
Atypical mental contents from infancy	Foreshortening of infantile amnesia period	Infantile influences on dreaming	Possible symptoms
– primordial emotions	o negative or positive o intense o unusual	o dreamed threats and other outsized emotions o nightmares	o phobias o anxiety
– infantile memory elements	o embodied (kinesthetic, vestibular) o preverbal o fragmentary, non-episodic	o typical dreams: flying, falling, chase, evil forces	o unusually good memory for childhood

Prematurely developed emotional processes	Adult-like memory	Adult-like dreaming	Possible symptoms
– fear expression, fear learning	o increased fear sensitivity	o poor dream affect regulation—including no affect	o affect distress o suicidal behavior
– fear extinction	o relapse-prone (vs. relapse-proof)	o reduced efficacy of REM extinction, REM and NREM emotion consolidation	o re-emergent primordial fears

Second, adversity-induced foreshortening of the infantile amnesia period may result in a greater number and variety of infantile memory elements influencing wake and dream processes later in life. Accordingly, individuals whose infantile amnesia period has been affected may report more frequent and more richly detailed memories of events that stem from ages 0–3 years than do others. These likely include memories of the adversities that led to infantile amnesia foreshortening in the first place, e.g., memories of separation, neglect, abuse, bullying, or other stressful experiences. Or, they may include affectively charged memories that are independent of adversity, e.g., early caregivers, a first pet, early dreams or milestones such as birthdays, and family festivities. Freud (126) claimed that dreams are replete with infantile memory elements, and he undertook voluminous descriptions of dreams and memory sources to illustrate the extent of this association. There have as yet appeared no alternative, easily applied methods for charting such early memory sources. Accurately tracking dream elements to infantile memory sources may well require protocols that are modeled on procedures for sampling early autobiographical memories. Training in introspective observation may also be needed.

For both waking and dreaming situations, memories arising prior to the normal infantile amnesia boundary are likely to be vivid, unusual, and qualitatively different from later childhood memories. Although phenomenological work on such unique early memories is needed, I suggest that at least two characteristics may distinguish them.

(1) *Episodic fragmentation*. Because the infantile amnesia period is pre-episodic, i.e., toddlers cannot normally form memories of specific events occurring in a particular time and place, memories from this period may be fragmented or incomplete—references to spatial context are especially likely to be missing (127) as these rely on maturation of the hippocampus.
(2) *Sensorimotor encoding*. Because infantile memories are largely preverbal and pre-symbolic, they may be encoded primarily in sensory form; motricity and body sensation are particularly important to early attempts at episodic memory (128) and might therefore be characteristic. The embodied features of memory have been described in the case of symptom formation among very young traumatized children, e.g., repetitive behaviors, trauma-specific fears (129).

In sum, foreshortening of the infantile amnesia period may lead—during both waking and sleep states—to abnormally better access to primordial emotions and infantile memory fragments; very likely, most such memories will comprise both components. While there is little phenomenological study of such memories, they are likely distinctive in being fragmented or incomplete, preverbal, pre-symbolic, and embodied. The present thesis is that these memories influence dreaming in a manner analogous to how they influence formation of the symptoms of mental illness. One effect may be an increase in memories of early-life dreams and nightmares (see later). The memories that are most likely to issue from the infantile amnesia period will likely reflect the dysphoric feelings and impressions of adverse events that led to the foreshortening effect, much like the flashbacks and nightmares of PTSD often reflect the traumatic experiences that led to the illness—as trauma replications, symbolic transformations, or emotional reactivations.

However, as the previous discussion should make clear, adversity-induced early-life changes may broaden access to a swath of memories beyond only negative events. Preserved infantile reminiscences may spare some emotionally positive or neutral features in addition to those linked to adversity. Animal studies (33) provide ample evidence for the post-adversity sparing of dysphoric memory traces but also give some indication that a more generalized sparing occurs. For example, some appetitive behaviors from the infantile period have been experimentally preserved, behaviors such as approaching a spout to drink sweetened milk (130). Similarly, human studies support the notion that infantile fear memories are preserved later in life but as yet similar evidence that positive or neutral memories are also preserved is still forthcoming (33). Some new supporting evidence is described later.

The next sections describe three types of evidence that converge to support the hypothesis that adult nightmares are associated with early childhood adversity and foreshortening of the infantile amnesia period.

#### Evidence from Clinical Histories of Nightmare Sufferers

Assessments of chronic nightmare sufferers provide evidence consistent with the notion that foreshortening of the infantile amnesia period increases access to an emotionally wide-ranging spectrum of one’s earliest memories. Clinical histories of nightmare sufferers described earlier (66) revealed that these patients remember their early childhoods “unusually well” (p. 67). Hartmann characterized his patients as intensely introspective, sensitive, interested in themselves and their childhoods, and possessing a psychological makeup somewhat unusual even at this early age. And although he did not consider infantile amnesia specifically in his theorizing about nightmare genesis, he did point squarely in the direction of this vital memory boundary:

It is true that we tend to forget or repress most events of the first four or five years of life; but I and other interviewers were impressed that these nightmare sufferers…exhibited less such repression than most of us … they were sometimes able to remember quite vividly scenes from their second, third, and fourth years. [(11, 66, 67), p. 106].

Hartmann’s probing for early trauma led him to a careful evaluation of the strength and quality of nightmare sufferers’ early memories, which inadvertently produced important evidence of increased access to memories from the infantile amnesia period in this population.^4^

#### Evidence from Early Dreams: Qualitative

Freud stipulated that infantile memories were readily identifiable in dreams possessing a typical content; he considered many such typical dreams as having relatively innocuous memory origins:

“There cannot be a single uncle who has not shown a child how to fly by rushing across the room with him in his outstretched arms, or who has not played at letting him fall by riding him on his knee and then suddenly stretching out his leg, or by holding him up high and then suddenly pretending to drop him.” “In after years they repeat these experiences in dreams; but in the dreams they leave out the hands which held them up …” (p. 375).

Furthermore, he considered such early experiences to constitute a source of dream anxiety: “*Childish ‘romping’*…*is what is being repeated in dreams of flying, falling, giddiness and so on; while the pleasurable feelings attached to these experiences are transformed into anxiety*.” [(126); pp. 375–376].

To investigate potential links between typical dream content and infantile experience, a convenience sample of early dream themes was examined for evidence of their resemblance to early childhood experiences (131). A total of 21,733 females (*M*_age_ = 25.1 ± 10.3 years) and 5,478 males (*M*_age_ = 25.7 ± 11.1 years) indicated on a 56-item checklist which dream themes they had experienced (as well as one additional “other” category). They estimated which of these dreams was (a) their most frequent, and (b) their earliest occurring; they also indicated (c) their age for the earliest occurring dream and estimated (d) their typical monthly nightmare recall using a 9-point scale.

**Figure 3:**
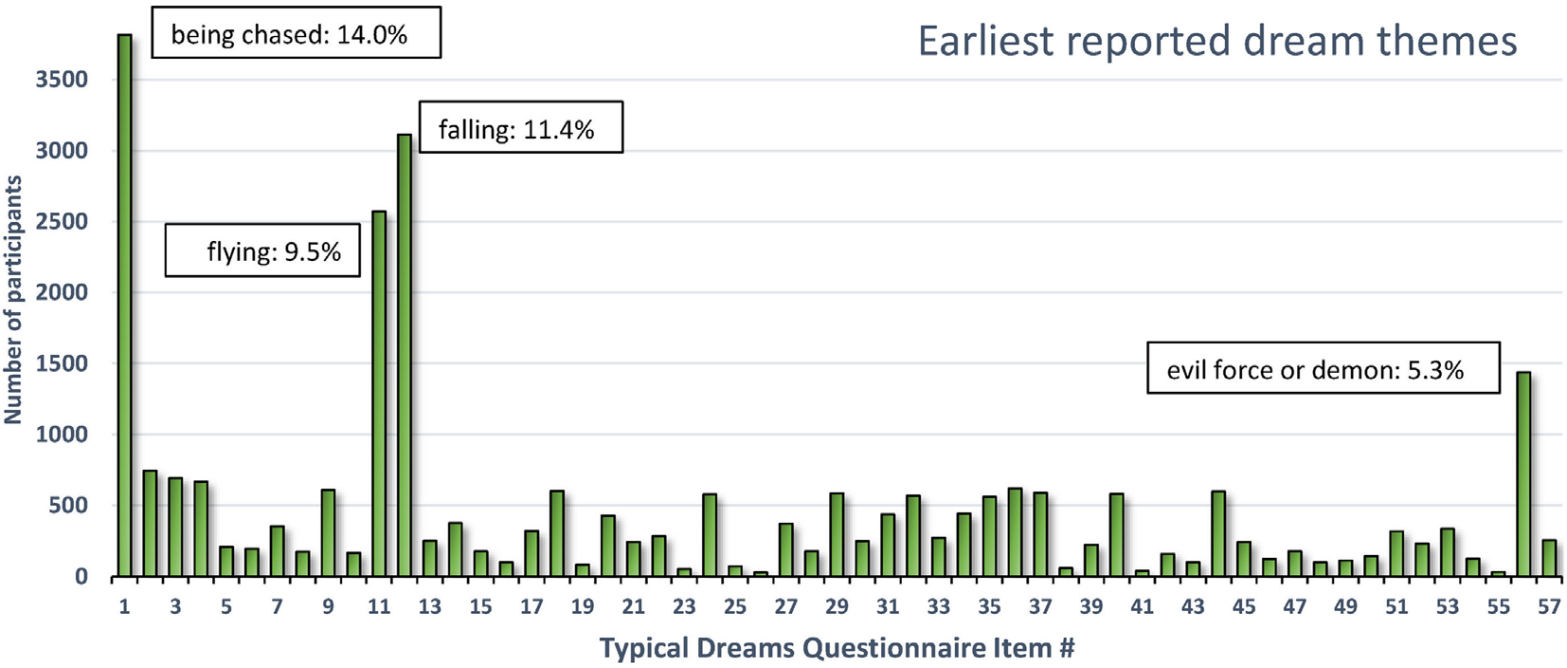
**Earliest reported typical dream themes for 27,211 participants**. Four themes accounted for 40.2% of all earliest themes reported (131).

As shown in Figure 3, the four most commonly selected earliest dreams accounted for 40.2% (10,940/27,211) of all selected dreams; in decreasing prevalence these were items *1-being chased* (14.02% or 3,816), *12-falling* (11.44% or 3,113), *11-flying or soaring through the air* (9.45% or 2,572), and *56-encountering a kind of evil force or demon* (5.29% or 1,439). All of the 53 other dreams (including *57-other*) individually accounted for less than 3% of the total number.

The four dreams most commonly identified as occurring earliest in life are readily interpretable as deriving from infantile experiences. The themes of falling and flying singled out by Freud were in fact the second and third most prevalent earliest dreams. Although these themes may well originate in early play experiences (126), they may also reflect more problematic early situations. Falling during early childhood is common (e.g., down stairs, from a bed, from a changing table or furniture, from a playground device, etc.) and can cause serious injuries (132). In one epidemiological study of 0- to 4-year olds (133), the annual rate of injury hospitalization and death from falls was twice the rate of the next most common cause (poisoning).

Dreams of being chased and demonic forces were not considered in detail by Freud but both reflect the common occurrence of threats in dreams (18) and nightmares (134) as if primitive “fight or flight” reactions tend to shape the content. The chase dream is one of the most cross-culturally constant themes (135–138), the most common nightmare theme [(67), p. 60] and the most prevalent recurrent dream theme from both childhood (41.5%) and adulthood (14.6%) (139). Accordingly, the game of chasing and being chased is very popular among young children. Even infants as young as 4 months are adept at visually recognizing chasing, possibly because it constitutes part of an innate, survival-based, action schema (140). It is feasible that early adverse experiences in which chase figures prominently trigger the occurrence of chase and threat dreams.

Altogether, this brief survey of early dream remembrances reveals that a limited number of themes accounts for most earliest recalled dreams. These themes—falling, flying, being chased, and evil forces—appear to reflect early childhood experiences of play (e.g., flying) but probably also adverse experiences such as falling or being chased. These may even reflect adverse experiences that led to a foreshortening of the infantile amnesia period to begin with.

#### Evidence from Early Dreams: Quantitative

The current model stipulates that early memories should be more available to individuals who have undergone early adverse experience, acceleration of the infantile amnesia boundary and, thus, who report more frequent nightmares as adults. Consequently, the model would predict that frequent nightmare sufferers more often remember dreams that are dated to earlier in life than do individuals without frequent nightmares. More specifically, the model would predict that frequent nightmare sufferers remember dreams that occurred prior to the end of the infantile amnesia period (3.5 years).

This hypothesis was tested quantitatively using the same responses described above and in two steps (131). First, current recall of nightmares (log-transformed) was assessed relative to participants’ ages when they had their earliest dream. Second, nightmare recall was assessed for participants whose earliest recalled dream was either positive or negative in tenor. It was expected that in both cases frequent nightmare sufferers would recall dreams from earlier ages than would controls.

##### Nightmares As a Function of Age of Earliest Dream

**Figure 4:**
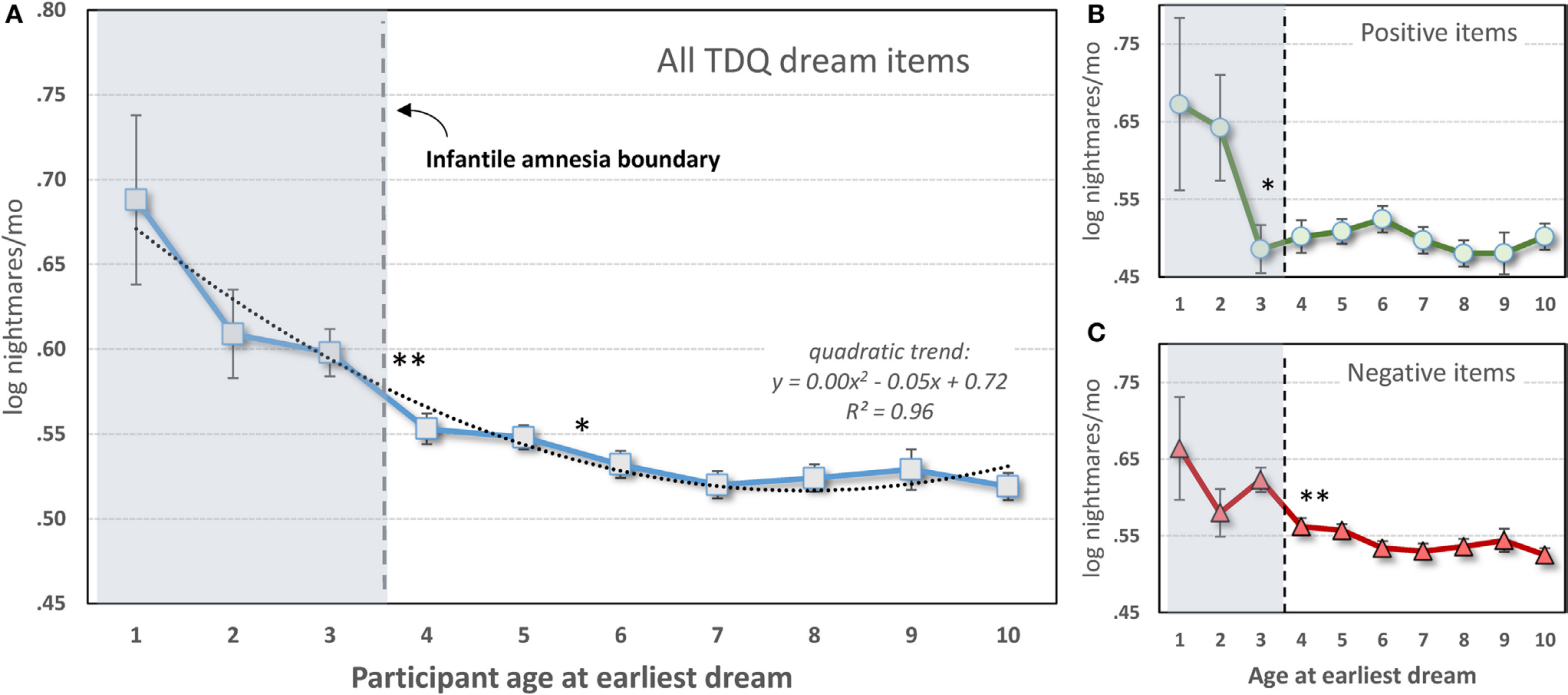
**Participant-estimated monthly nightmare recall for earliest typical dreams occurring at different ages**. **(A)** All 56 items of the Typical Dreams Questionnaire (TDQ) included (*n* = 17,014); **(B)** 12 positive TDQ items (*n* = 3,366); **(C)** 32 negative TDQ items (*n* = 11,868). In all three cases, current nightmares are more frequent among participants whose earliest memories fall within the infantile amnesia period, which normally ends at 3.5 years of age (vertical hashed lines). Significant differences are shown only for comparisons of adjacent ages: **p < 0.01; *p < 0.05 [from Ref. (131)].

Only 17,014 participants (3,535 males; 13,479 females) from the data set who specified an earliest dream occurring at age 10 or younger were included. As shown in Figure 4A, a one-way ANOVA with 10 levels of Dream-Age (1–10 years) as independent variable and current (log) nightmare recall as dependent variable revealed a highly significant main effect (*F*_9,17004_ = 6.08, *p* < 0.0000001) showing that earlier recalled dreams were associated with frequent current nightmares. Contrasts revealed significant differences between ages 3 and 4 and ages 5 and 6. All of ages 1–3 differed from all of ages 4–10.

These results are striking support for the hypothesis. Participants whose early dream memories fell within the infantile amnesia window had much higher current rates of nightmares than did participants who had more normal memory access outside of this window—and this was independent of current age. The significant change from ages 3 to 4 is particularly noteworthy as 3.5 years is when infantile amnesia normally ends and adult-like memory processing begins. And although there were no significant differences in nightmare frequency between ages 1 and 3, there is a clear graded relationship between the two measures.

##### Nightmares As a Function of Early Dream Valence

The foreshortening effect for positive dreams was assessed by repeating the analysis only with participants who identified 1 of 12 Typical Dreams Questionnaire (TDQ) dream themes with an emotionally positive valence, e.g., *eating delicious foods, flying*, or *seeing an angel*. A largely similar pattern of results was found (Figure 4B). Participants recalling earlier positive dreams again reported more current nightmares, but there was now a significant breakpoint between ages 2 and 3. Age 2 differed from all later years (*p* < 0.05) except ages 5 and 6 for which there were trends. Age 1 had a low sample size (12) and only differed marginally from later ages.

The effect for negative dreams was assessed by repeating the analysis with participants who recalled one of 32 emotionally negative TDQ items, e.g., being *chased, physically attacked, killed*, or *smothered*. The same pattern was again found (Figure 4C), i.e., nightmare frequency was higher for recall at age 3 than at age 4 and marginally higher at age 5 than at age 6. *Post hoc* tests again revealed differences between ages 1 and 3, but not age 2, and all later ages, specifically.

These results together support the additional expectation that foreshortening of the infantile amnesia period is independent of the dream memory’s emotional valence, i.e., that a generalized increase in access to early memories is provoked. Not only does this finding support Hartmann’s early observation that nightmare sufferers have “unusually good” memories for early childhood but it may also explain why frequent nightmare sufferers also more often report a range of positive dream experiences (e.g., flying dreams, lucid dreams), are more frequently endowed with more creative skills (141), and display broader than normal access to emotional semantic networks (142). Increased access to a greater range of early memories may set the stage for development of both maladaptive and adaptive cognitive abilities in adulthood.

#### Evidence from Behavioral and Animal Studies

Cognitive phenomenological evidence that memories from the infantile amnesia period can influence later cognitive activity cannot, understandably, be replicated with non-human animal studies. Nonetheless, animal studies do provide converging behavioral and neurophysiological support for the stress acceleration thesis. There are two main types of evidence: results demonstrating that (1) learning during the infantile amnesia period continues to influence later behavior—even if that learning is forgotten and (2) adversity accelerates the transition from infantile to adult modes of emotional learning.

##### Infantile Learning Continues to Influence Behavior

Evidence for continuing effects of infantile learning on future behavior [reviews in Ref. (33, 91, 143)] consists in demonstrations that fear conditioning during the infantile period alters the neural mechanisms of future emotional learning. This continuing effect is frequently referred to as a memory “trace” or “engram” that influences future behavior (91). For example, one review (143) concludes that “… *early memories leave at least a partial trace that continues* to influence later functioning despite not being explicitly recalled …” (p. 136).

This type of covert early influence is illustrated in rodent studies that assess the effects on future learning of a memory acquired during infancy but then forgotten. One study (144) used the fact that, in adult animals, first-time task learning is dependent on activation of *N*-methyl-d-aspartate (NMDA) receptors (145) but later task relearning is not (146). Li and Richardson (144) demonstrated that this change from NMDA dependence to NMDA independence remains true even if very young rats that have forgotten the initial fear conditioning task are retrained on it in adulthood. The early learning changed the neurobiology of later learning even though the learned behavior was forgotten.

In a second study (147), infant rats exposed to inescapable shock in one multisensory context did not fear that context as adults but did express stronger fear learning to a different context. Again, the neurocircuitry of fear learning was altered even though the learning originating in the infantile amnesia period was forgotten. Poulos et al. (147) consider this augmented fear conditioning to be analogous to those types of PTSD patients who do not remember their precipitating traumatic event (e.g., asphyxiation with amnesia) yet exhibit a greater propensity for developing phobias and expressing reactivity to other emotional stimuli (148, 149).

Such studies have also revealed neurobiological changes that accompany the behavioral changes. In an experiment similar to that of Li and Richardson (144), Kim et al. (150) showed that, despite forgetting a task on a behavioral level, infant rats exhibited persisting amygdala activity (mitogen-activated protein kinase phosphorylation) that reflected the early-life learning. Similarly, in the Poulos et al.’s (147) study, persistence of the infantile trace was accompanied by increased expression of amygdala glucocorticoid receptors and atypical (2-peak) circadian rhythms of basal corticosterone. Numerous other neural changes are detailed in the review papers cited above. Such long-term effects are akin to recently discovered persisting health effects presaged by early adversity, such as adversity at 1.5 years predicting increased levels of C reactive protein at age 15 (151), or sexual abuse at ages 3–5 (vs. abuse in adolescence) being associated with increased Epstein–Barr virus antibodies in adulthood (152).

##### Adversity Accelerates Transition from Infantile to Adult Emotional Learning

Much evidence for the accelerated infantile amnesia boundary points to early adversity changing two principal types of emotional processes: fear retention and fear extinction. In the case of fear retention, animals exposed to early adversity retain fear memories much longer than usual [review in Ref. (143)]. For example (153), with maternal separation used as an adverse rearing condition, infant rats were given trials pairing a noise (as CS) and a shock (as US) and then tested for their fear of the noise (behavioral freezing) 1 or 10 days later. Maternally separated pups learned the association as well as did standard-reared pups—both expressed fear 1 day later—but the standard-reared pups forgot the association 10 days later, expressing little CS-elicited freezing, whereas maternally separated pups maintained high levels of freezing at 10 days and even at 30-day follow-up. Similar effects were observed if early adversity was induced by administering the stress hormone corticosterone to mothers rearing the pups. Similar effects were also produced (83) when the adverse condition consisted of an acute stressor (24-h maternal separation) rather than a chronic one (3-h/day separation, postnatal days 2–14). Li et al. (143) suggest that their findings help explain why early adversity predicts later anxiety disorders [e.g., Ref. (154)]; like the rat pups, anxious individuals may retain memories of early adverse experiences much longer than normal. The same may be true of nightmare sufferers, for whom the memories are expressed mainly during dysphoric dreams.

For fear extinction, young animals exposed to adverse conditions shift prematurely from a *relapse-resistant* (infantile) mode of extinction learning to a *relapse-prone* (adult) mode [review in Ref. (155)]. Thus, adversity-exposed animals will more easily retain a learned fear response even after extinction training, e.g., if a reminder of the fear is given, rather than rapidly erasing the fear memory as a control animal would do. To illustrate, two groups of infant rat pups, one maternally deprived and one not, both learned a fear retention task—shock-associated freezing—equally well and both were trained to extinguish this response equally effectively in a given context; the maternally separated group showed a return of fear when tested in a new context whereas the non-separated group did not; this relapse effect continued at 14-day follow-up (83).

Studies such as these demonstrate the cross-species generality of stress acceleration effects. Both acute and chronic early-life adversities reliably induce a premature transition from infant to adult-like expression of fear memory processes; stressed animals cease to exhibit normal infantile amnesia in favor of a more adult-like pattern of fear retention and relapse-prone fear extinction. While it remains to be clarified how such acceleration effects lead to long-term dysfunction, Cowan et al. (83) suggest that a disruption of differential developmental timing may be a factor; early maturation of some emotional processes creates competition for other neural systems that are developing concurrently (156), in other words, the *behavioral neotony* of immature individuals may be adversely affected.

#### Evidence from Changes in Sleep Architecture

The notion that stress acceleration contributes to nightmare pathology would be bolstered by parallel evidence that early adversity accelerates sleep-related mechanisms implicated in nightmare etiology—especially as these mechanisms manifest around the time of the infantile amnesia boundary. Evidence for such changes is at present slim, although several areas of study may be mined for corroborating findings: descriptive studies of sleep architecture in infants and toddlers, infant sleep in relation to attachment security, and animal studies of early adversity effects on sleep to name a few. The following preliminary review supports the possibility that some markers of sleep’s emotional memory and regulation functions, including NREM sleep spindles and REM sleep proportion, change abruptly around the end of the infantile amnesia period, thus paralleling the developmentally normal memory and dreaming changes described earlier. Some evidence also suggests that adversity prior to the infantile amnesia boundary leads to accelerated development of these sleep markers as it does with fear memory processes.

##### NREM Sleep and Sleep Spindles

NREM sleep spindles, primarily stage 2 (N2) sleep spindles, reflect memory plasticity mechanisms [reviews in Ref. (157, 158)], especially the consolidation of new learning [reviews in Ref. (159, 160)]. For example, increases in spindle density follow improvements on a motor task in a graded fashion; performance gains correlate positively with the rate of spindle expression during intervening sleep (161). This is the case even for children (36–67 months) trained on a visuospatial location task and allowed to nap; sleep spindle density correlates positively with degree of pre- to post-nap task improvement (162).

Sleep spindle density is also associated with the consolidation of emotional memories and with development of emotion regulation. In fact, increasing spindle density pharmacologically improves memory for emotional stimuli (163). In 5-year olds, spindle density is associated with favorable emotion/behavior patterns, such as less internalizing behavior and more prosocial behavior (164). Spindle density also correlates differentially with positive and negative coping strategies on the MacArthur Story Stem Battery, i.e., negatively with use of denial/avoidance and positively with use of positive emotions (165). Critically, spindle density correlates positively with dream and nightmare recall (166) and, compared with controls, nightmare sufferers have more fast spindles and correlations between spindles and anxiety (167).

**Figure 5:**
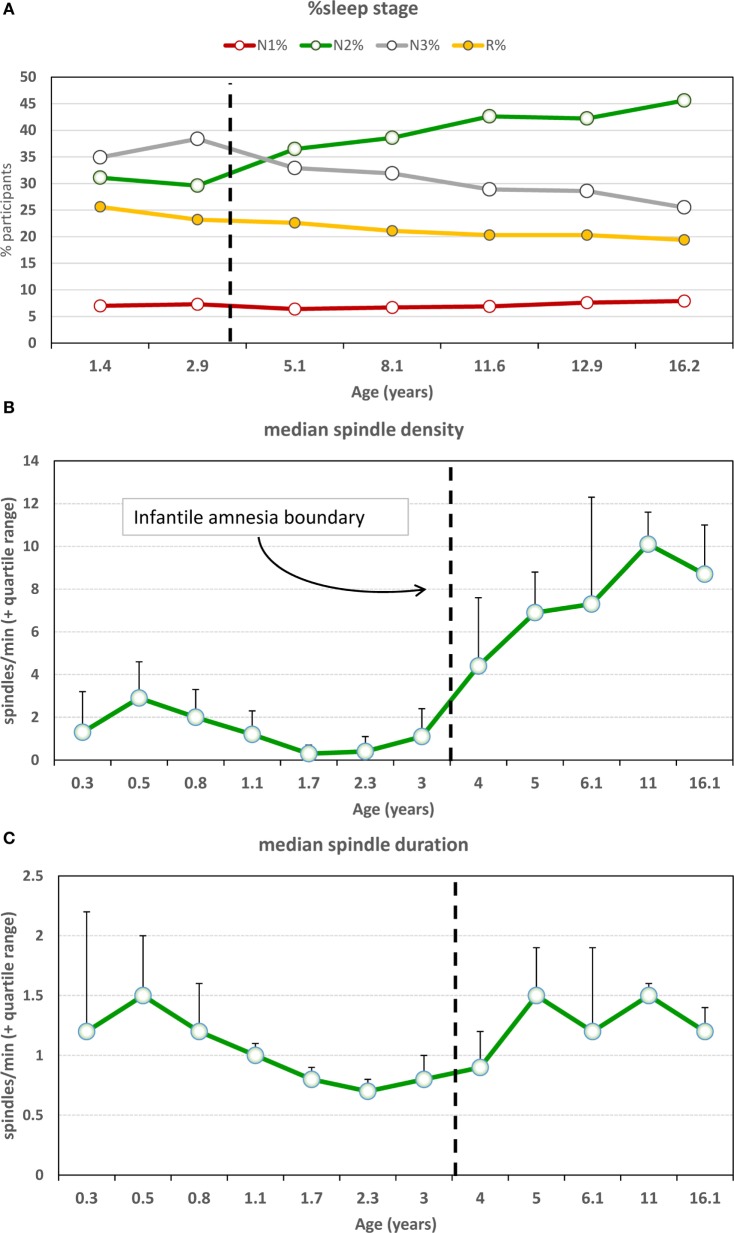
**(A)** Sleep stage percentages and sleep spindle characteristics as a function of age. There is a marked increase in N2% and simultaneous decrease in N3% at the infantile amnesia boundary [from Ref. (168)]. **(B)** Spindle density shows a clear and persistent increase across the infantile amnesia boundary, whereas **(C)** spindle duration increases from ages 4 to 5 [from Ref. (168)]. N2 sleep and spindle density are commonly associated with sleep-related memory plasticity changes and have been linked to cognitive capacities, e.g., IQ.

Important developmental changes also characterize the expression of sleep spindles near the infantile amnesia boundary. As shown in Figures 5A,B, sudden persistent increases in both N2% sleep and N2 spindle density occur between ages 3 and 4 (168). There is also an increase in spindle duration slightly later, from 4 to 5 years (Figure 5C). Evidence is still scant about whether early adversity accelerates these switches to “adult-like” spindle functioning as the SAH-NM hypothesis would predict; however, one study (169) shows that inter-spindle interval decreases and spindle duration increases after head trauma in infants <3.5 years.

##### REM Sleep: REM%, REM Latency, and REM Theta

REM sleep or *active sleep*, its physiological precursor in infants, is the predominant sleep stage in newborns, comprising on average 60% (range: 55–80%) of total sleep (170). Normally, REM% decreases through infancy; a comparison of 2- and 5-year olds found reductions in both REM minutes (175–140) and REM% (29–24%) (171). This REM decrease may be accelerated by early adversity. A review of eight relevant studies (172) revealed that maternal separation produced REM sleep inhibition or disappearance altogether and longer REM latencies. To illustrate, in one study (173), 10 pigtail monkeys maternally separated at age 26 weeks showed decreases in REM sleep from baseline (M = 90 min) on all 4 separation days (M = 21, 36, 48, and 46 min) but a robust rebound on 3 reunion days (M = 88, 95, and 89 min). Also, REM latency increased from baseline (M = 64 min) to a 4-day mean of 172.7 min during separation followed by a return to near-baseline (M = 102, 80, and 77 min) on reunion days. Such demonstrations of immediate negative influences of adversity on REM sleep suggest—but do not prove—that with chronic maternal separation, REM sleep parameters may be permanently altered.

More recent findings concerning chronic adversity, although mixed, do indicate such a permanent change—but in a direction opposite to that for acute separation. Some studies show that early adversity produces permanent changes in baseline REM sleep, e.g., increased REM% (174, 175), more REM episodes with more theta oscillations (175), shorter REM latency, and longer REM duration, as well as reduced SWS (176). One study (175) showed parallel increases in fear memory retention and REM sleep, including increased neuronal replay (theta synchronization in REM) in hippocampal, amygdala, and medial prefrontal circuits. However, other work suggests that permanent changes may only be detected when the animal’s REM sleep is later challenged by stress. For example (177), female rats exposed to either long maternal separation (LMS) or brief maternal separation as infants had similar baseline sleep as adults; however, after being stressed (1 h in cold chamber), LMS rats alone showed a REM sleep increase. Other work using chronically maternal separation (178) combined with chronic mild stressors in adulthood (tilted cage, wet bedding, social threat, etc.) produced similar results: more REM sleep, REM episodes and NREM episodes ending in REM sleep, and less REM theta. In brief, early chronic adversity permanently changed how REM sleep responded to later stress challenges; its regulatory functionality may have been altered to become more reactive. This reactivity may include an increased likelihood of nightmares.

In fact, in human participants similar changes in REM sleep have been documented; changes in dream emotion have also been found. In one study (179), women with irritable bowel syndrome (IBS) and a history of abuse/neglect had higher REM% sleep than did women without abuse/neglect, either with or without IBS; groups did not differ on other PSG measures. In a second study (180), participants with anxious attachment styles had shorter REM sleep latencies and more frequent REM dreams containing aggression and self-denigrating themes than did participants with secure attachment.

Thus, although acute adversity tends to reduce the amount of REM sleep, chronic adversity either increases it or alters REM reactivity such that it is more likely to increase when confronted with sources of stress. While more research is needed to resolve discrepancies with animal studies, these findings do suggest a parallel with the proposed origins of nightmares, i.e., chronic early adversity may lead either to recurrent lifelong nightmares or render individuals more sensitive to ongoing sources of stress, which in turn trigger nightmares.

In sum, several NREM and REM sleep attributes associated with sleep’s emotional memory and regulation functions change abruptly around the end of the infantile amnesia period, thus mirroring changes documented for the waking state. Some evidence supports the additional notion that these changes may be accelerated by early adversity, again in parallel with waking changes in emotional memory. Taken together with evidence of changes in early dream production and in the recall of early dreams around the infantile amnesia boundary, there is converging support for the central tenets of the stress acceleration hypothesis of nightmares.

### Neural Effects of Early Adversity

**Figure 6:**
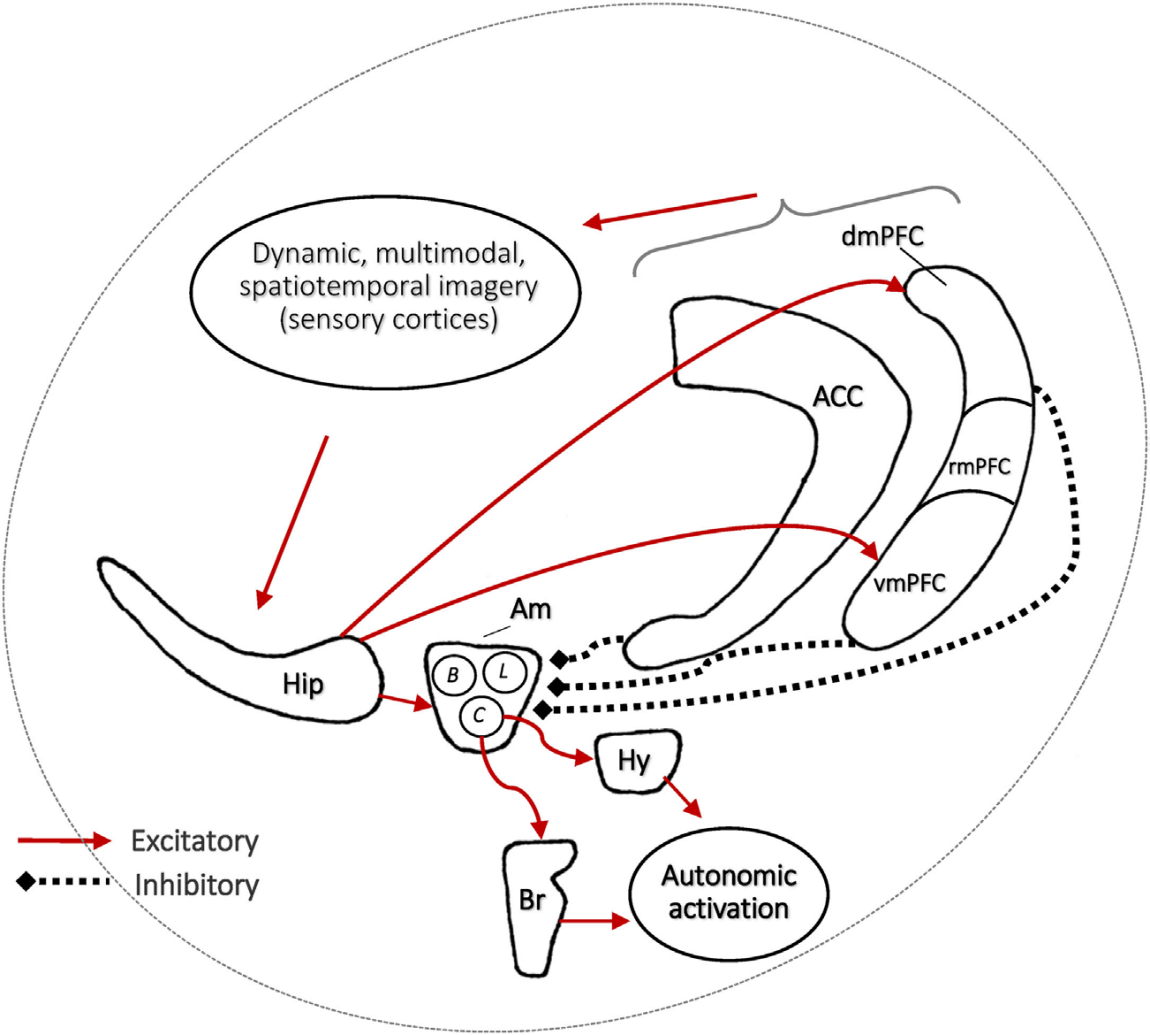
**The Nightmare-Affect Network Dysfunction systems-level model of fear memory processes showing only the main connections thought to influence fear memory production and extinction during REM sleep dreaming**. The model focuses on the amygdala (Am), hippocampus (Hip), anterior cingulate cortex (ACC), and medial prefrontal cortex (mPFC)—especially the vmPFC and dmPFC segments. It also displays the influence of prefrontal cortex on hippocampus *via* sensory cortices (visual, auditory, somatosensory) in the production of dynamic multimodal and spatiotemporally veridical imagery as well as the generation of amygdala-provoked autonomic activation. This network is hypothesized to support a REM sleep and dreaming function of producing and maintaining fear extinction (safety memories) and of preventing fear from returning (renewal, spontaneous recovery). Although the vmPFC, dmPFC, and ACC interact with Am and Hip bidirectionally for multiple affective and decision-making functions, the figure shows only pathways relevant to their inhibitory regulation of Am output by cortical and hippocampal mediation to facilitate fear extinction and prevent fear renewal by safety memory contextualization. Am stimulates Br and Hy to activate autonomic correlates of fear imagery, e.g., tachycardia. Dysfunction in the network produces nightmares and may be due to Am over-activation or failure of hippocampal/PFC downregulation of fear during imagery formation. However, it remains unknown if nightmares themselves are pathological or reflect an active mechanism of extinction or contextualization. vmPFC, ventromedial PFC; rmPFC, rostromedial PFC, dmPFC, dorsomedial PFC; C, central nucleus; B, basolateral nucleus; L, lateral nucleus; Br, brainstem; Hy, hypothalamus.

The literature covering adversity-induced changes in neural development in humans (34, 181, 182), non-human primates (183), and rodents (184) is too extensive to review here. Research detailing the neural changes underlying stress acceleration effects, in particular, has been reviewed by several authors (9, 185–187) and will be discussed only selectively in the present context. Findings showing that adversity triggers changes to a fear memory and extinction circuit is of particular interest to the present approach because the regions implicated in the reviewed work overlap substantially with components of a model previously proposed as a neurocognitive explanation for nightmare pathology (5, 16). This model, a modified version of which is referred to here as the *Nightmare*-*Affect Network Dysfunction* (NM-AND) model, is illustrated in Figure 6. The substantial overlap between the stress acceleration findings and the NM-AND model allows incorporation of the latter into a wider, more developmentally coherent framework and opens nightmare research to a greater number of hitherto unexamined questions.

The fear extinction function proposed by the NM-AND model to occur during sleep is similar in some respects to the affective contextualization function proposed for the default mode network (188), which partially overlaps (vmPFC, ACC) the NM-AND network and which, intriguingly, may also be active during dreaming (189–192). Although other brain regions have been shown to support fear memory processes since first publication of the AND model, and although the circuits of the model are now acknowledged to be implicated in many emotion regulation functions, there is still a consensus that the Am, Hip, vmPFC, and ACC are central to fear learning [review in Ref. (193, 194)] and, especially, fear extinction [reviews in Ref. (187, 188, 195)]. New research implicates vmPFC in the learning of *safety memories* by the inhibition of fear responses previously linked to danger signals in the Am (196) and implicates a dmPFC–hippocampus circuit that controls fear renewal by modulating Am output according to differing contexts (197). Children exposed to early adversity may be more dependent on the latter circuit (198). New research also links mPFC to the production of multimodal imagery (199–203) and spatiotemporal orientation (204, 205).

## Conclusion

Substantial progress has been made in delineating the immediate and long-term neural changes wrought by adversity. Adversity can deleteriously affect the future physical and mental health of a child, thereby increasing their risk for many illnesses, including nightmares. A major effect of early adversity is to accelerate maturation of emotional behaviors and neurocircuits during the developmentally sensitive, infantile amnesia period. This may increase risk for developing nightmares by (1) foreshortening the duration of the normal infantile amnesia period, such that normally forgotten, childhood memories dating to earlier than 3.5 years of age continue to influence later emotions, cognitions and behavior—including dreaming and (2) altering the normal emotion regulation circuitry of both waking and sleep such that there is exaggerated fear learning and relapse-prone fear extinction. The neural changes wrought by adversity are particularly noticeable in a network of brain regions which includes the amygdala and its modulating circuits, the hippocampus, mPFC, and ACC.

While converging evidence reviewed here supports these conclusions, there are still important gaps in our understanding of how early adversities alter the neural and cognitive processes of wakefulness and sleep. Even if it is clear that childhood adversity produces immediate structural changes in amygdala and its modulating regions and that, in adulthood, amygdala reactivity is chronically elevated while mPFC regulatory control is chronically ineffective (9), it remains to be explained how the former changes lead causally to the latter. The stress acceleration approach provides an open framework within which longitudinal theories of symptom etiology can be elaborated. The present SAH theory of nightmares is one such endeavor.

## Author Contributions

TN was solely responsible for the development and rendering of the manuscript.

## Conflict of Interest Statement

The author declares that the research was conducted in the absence of any commercial or financial relationships that could be construed as a potential conflict of interest.

## Footnotes

^1^Freud considered many memory traces from the infantile period to consist of inchoate sexual feelings and proposed that these memories were transformed into unreliable “screen memories” so as to avoid the anxiety that the infantile memories might evoke. In a related vein, psychoanalytic scholar Ernest Jones proposed that nightmares originate in repressed sexual desires (97).

^2^Distinguishing nightmares (a REM sleep phenomenon) from sleep terrors (a non-REM sleep phenomenon) without the benefit of polysomnographic recordings is problematic, especially among preschool and preverbal children. However, in light of recent evidence [e.g., Ref. (119)] that somnambulistic episodes (of which sleep terrors are one type) are associated with emotionally vivid dream content as frequently (80%) as dreams are associated with REM sleep awakenings (80%), and evidence that dreaming in both REM and non-REM sleep are produced by the same dream-producing mechanism, it is likely that adversity-induced changes to this dream mechanism will influence both REM sleep nightmares and non-REM sleep terrors, in addition to other disturbed forms of dreaming (e.g., sleep paralysis, terrifying hypnagogic hallucinations). Thus, the SAH-NM approach may help explain the pathophysiology of sleep terrors including, especially, why REM sleep is disturbed in this non-REM sleep disorder (120).

^3^The intense nightmarish mentation accompanying sleep terrors often encapsulates primordial emotions of the type described for vegetative systems such as thirst, the hunger for air or food, pain, or sexual arousal (121). As sleep terrors are also influenced by early adversity, the stress-acceleration hypothesis of nightmares may account for the development of sleep terrors as well.

^4^Hartmann’s in-depth assessment of lifelong nightmare sufferers was an important contribution to developing his notion of “thin boundaries,” which he subsequently validated as a basic personality construct. The present analysis raises the intriguing possibility that breaching of the *infantile amnesia boundary* among nightmare sufferers may inadvertently have influenced Hartmann’s conceptualization of this more general personality construct.

## References

[1] American Academy of Sleep Medicine. International Classification of Sleep Disorders – Third Edition (ICSD-3). Darien, IL: American Academy of Sleep Medicine (2014).

[2] American Psychiatric Association. Diagnostic and Statistical Manual of Mental Disorders, 5th Edition (DSM-5). Washington, DC: American Psychiatric Publishing (2013).

[3] Davis M The role of the amygdala in conditioned and unconditioned fear and anxiety. In: Aggleton JP, editor. The Amygdala. Oxford: Oxford University Press (2000). p. 213–87.

[4] LeDoux JE Emotion circuits in the brain. Annu Rev Neurosci (2000) 23:155–84. 10.1146/annurev.neuro.23.1.155 10845062

[5] Levin R Nielsen TA. Disturbed dreaming, posttraumatic stress disorder, and affect distress: a review and neurocognitive model. Psychol Bull (2007) 133:482–528. 10.1037/0033-2909.133.3.482 17469988

[6] Nielsen T Disturbed dreaming as a factor in medical conditions. 5th ed In: Kryger M Roth T Dement WC, editors. Principles and Practice of Sleep Medicine. New York, NY: Elsevier (2011). p. 1116–27.

[7] Coolidge FL Segal DL Coolidge CM Spinath FM Gottschling J. Do nightmares and generalized anxiety disorder in childhood and adolescence have a common genetic origin? Behav Genet (2010) 40:349–56. 10.1007/s10519-009-9310-z 19902346

[8] Klůzová Kráčmarová L Plháková A Nightmares and their consequences in relation to state factors, absorption, and boundaries. Dreaming (2015) 25:312–20. 10.1037/a0039712

[9] Callaghan BL Tottenham N The neuro-environmental loop of plasticity: a cross-species analysis of parental effects on emotion circuitry development following typical and adverse caregiving. Neuropsychopharmacology (2016) 41:163–76. 10.1038/npp.2015.204 26194419 PMC4677125

[10] Nielsen T Levrier K Montplaisir J Dreaming correlates of alexithymia among sleep-disordered patients. Dreaming (2011) 21:16–31. 10.1037/a0022861

[11] Hartmann E The Nightmare: The Psychology and the Biology of Terrifying Dreams. New York: Basic Books (1984).

[12] Nielsen T Powell RA. Dreams of the Rarebit Fiend: food and diet as instigators of bizarre and disturbing dreams. Front Psychol (2015) 6:47. 10.3389/fpsyg.2015.00047 25741294 PMC4330685

[13] Nielsen T Carr M Nightmares and nightmare function. In: Kryger M Roth B Dement W, editors. Principles and Practice of Sleep Medicine (Vol. 6). New York: Elsevier (2016). p. 56–54.

[14] Fisher C Byrne J Edwards A Kahn E A psychophysiological study of nightmares. J Am Psychoanal Assoc (1970) 18:747–82. 10.1177/000306517001800401 4321827

[15] Walker MP van der Helm E. Overnight therapy? The role of sleep in emotional brain processing. Psychol Bull (2009) 135:731–48. 10.1037/a0016570 19702380 PMC2890316

[16] Nielsen T Levin R. Nightmares: a new neurocognitive model. Sleep Med Rev (2007) 11:295–310. 10.1016/j.smrv.2007.03.004 17498981

[17] Revonsuo A The reinterpretation of dreams: an evolutionary hypothesis of the function of dreaming. Behav Brain Sci (2000) 23:877–901. 10.1017/S0140525X00004015 11515147

[18] Valli K Revonsuo A. The threat simulation theory in light of recent empirical evidence: a review. Am J Psychol (2009) 122:17–38. 19353929

[19] Cronholm PF Forke CM Wade R Bair-Merritt MH Davis M Harkins-Schwarz M Adverse childhood experiences: expanding the concept of adversity. Am J Prev Med (2015) 49:354–61. 10.1016/j.amepre.2015.02.001 26296440

[20] Teicher MH Parigger A. The ‘Maltreatment and Abuse Chronology of Exposure’ (MACE) scale for the retrospective assessment of abuse and neglect during development. PLoS One (2015) 10:e0117423. 10.1371/journal.pone.0117423 25714856 PMC4340880

[21] Wade R Jr Shea JA Rubin D Wood J. Adverse childhood experiences of low-income urban youth. Pediatrics (2014) 134:e13–20. 10.1542/peds.2013-2475 24935995

[22] Wildeman C Emanuel N Leventhal JM Putnam-Hornstein E Waldfogel J Lee H. The prevalence of confirmed maltreatment among US children, 2004 to 2011. JAMA Pediatr (2014) 168:706–13. 10.1001/jamapediatrics.2014.410 24887073 PMC5087599

[23] Carr M Solomon G Nielsen T Is nightmare disorder a sub-clinical form of PTSD? Sleep (2014) 37(Suppl):A271.

[24] Schreuder BJN Igreja V van Dijk J Kleijn W Intrusive re-experiencing of chronic strife or war. Adv Psychiatr Treat (2001) 7:102–8. 10.1192/apt.7.2.102

[25] Vanderkolk BA Fisler R Dissociation and the fragmentary nature of traumatic memories – overview and exploratory study. J Trauma Stress (1995) 8:505–25. 10.1007/BF02102887 8564271

[26] Wilmer HA, editor. Trauma and dreams. The Healing Nightmare: War Dreams of Vietnam Veterans. Cambridge, MA: Harvard University Press (1996). p. 85–99.

[27] Wittmann L Schredl M Kramer M. Dreaming in posttraumatic stress disorder: a critical review of phenomenology, psychophysiology and treatment. Psychother Psychosom (2007) 76:25–39. 10.1159/000096362 17170561

[28] Phelps AJ Forbes D Hopwood M Creamer M. Trauma-related dreams of Australian veterans with PTSD: content, affect and phenomenology. Aust N Z J Psychiatry (2011) 45:853–60. 10.3109/00048674.2011.599314 21859279

[29] Gauchat A Seguin JR McSween-Cadieux E Zadra A. The content of recurrent dreams in young adolescents. Conscious Cogn (2015) 37:103–11. 10.1016/j.concog.2015.08.009 26366465 PMC4851546

[30] Domhoff GW Finding Meaning in Dreams. A Quantitative Approach. New York, NY: Plenum (1996).

[31] Germain A Nielsen TA. Sleep pathophysiology in posttraumatic stress disorder and idiopathic nightmare sufferers. Biol Psychiatry (2003) 54:1092–8. 10.1016/S0006-3223(03)00071-4 14625152

[32] Gunnar MR Hostinar CE Sanchez MM Tottenham N Sullivan RM. Parental buffering of fear and stress neurobiology: reviewing parallels across rodent, monkey, and human models. Soc Neurosci (2015) 10:474–8. 10.1080/17470919.2015.1070198 26234160 PMC5198892

[33] Madsen HB Kim JH. Ontogeny of memory: an update on 40 years of work on infantile amnesia. Behav Brain Res (2016) 298:4–14. 10.1016/j.bbr.2015.07.030 26190765

[34] Teicher MH Samson JA. Annual research review: enduring neurobiological effects of childhood abuse and neglect. J Child Psychol Psychiatry (2016) 57:241–66. 10.1111/jcpp.12507 26831814 PMC4760853

[35] Anda RF Brown DW Dube SR Bremner JD Felitti VJ Giles WH. Adverse childhood experiences and chronic obstructive pulmonary disease in adults. Am J Prev Med (2008) 34:396–403. 10.1016/j.amepre.2008.02.002 18407006 PMC8214869

[36] Brown DW Anda RF Felitti VJ Edwards VJ Malarcher AM Croft JB Adverse childhood experiences are associated with the risk of lung cancer: a prospective cohort study. BMC Public Health (2010) 10:20. 10.1186/1471-2458-10-20 20085623 PMC2826284

[37] Kalmakis KA Chandler GE. Health consequences of adverse childhood experiences: a systematic review. J Am Assoc Nurse Pract (2015) 27:457–65. 10.1002/2327-6924.12215 25755161

[38] Nusslock R Miller GE Early-life adversity and physical and emotional health across the lifespan: a neuroimmune network hypothesis. Biol Psychiatry (2015) 80:23–32. 10.1016/j.biopsych.2015.05.017 26166230 PMC4670279

[39] Su S Jimenez MP Roberts CT Loucks EB. The role of adverse childhood experiences in cardiovascular disease risk: a review with emphasis on plausible mechanisms. Curr Cardiol Rep (2015) 17:88. 10.1007/s11886-015-0645-1 26289252 PMC4941633

[40] Tung J Archie EA Altmann J Alberts SC. Cumulative early life adversity predicts longevity in wild baboons. Nat Commun (2016) 7:11181. 10.1038/ncomms11181 27091302 PMC4838827

[41] Brown DW Anda RF Tiemeier H Felitti VJ Edwards VJ Croft JB Adverse childhood experiences and the risk of premature mortality. Am J Prev Med (2009) 37:389–96. 10.1016/j.amepre.2009.06.021 19840693

[42] Beards S Gayer-Anderson C Borges S Dewey ME Fisher HL Morgan C. Life events and psychosis: a review and meta-analysis. Schizophr Bull (2013) 39:740–7. 10.1093/schbul/sbt065 23671196 PMC3686461

[43] Bentall RP de Sousa P Varese F Wickham S Sitko K Haarmans M From adversity to psychosis: pathways and mechanisms from specific adversities to specific symptoms. Soc Psychiatry Psychiatr Epidemiol (2014) 49:1011–22. 10.1007/s00127-014-0914-0 24919446

[44] Green JG McLaughlin KA Berglund PA Gruber MJ Sampson NA Zaslavsky AM Childhood adversities and adult psychiatric disorders in the national comorbidity survey replication I: associations with first onset of DSM-IV disorders. Arch Gen Psychiatry (2010) 67:113–23. 10.1001/archgenpsychiatry.2009.186 20124111 PMC2822662

[45] Hillis SD Anda RF Dube SR Felitti VJ Marchbanks PA Marks JS. The association between adverse childhood experiences and adolescent pregnancy, long-term psychosocial consequences, and fetal death. Pediatrics (2004) 113:320–7. 10.1542/peds.113.2.320 14754944

[46] LeardMann CA Smith B Ryan MA. Do adverse childhood experiences increase the risk of postdeployment posttraumatic stress disorder in US Marines? BMC Public Health (2010) 10:437. 10.1186/1471-2458-10-437 20659342 PMC2916906

[47] Baiden P Fallon B den Dunnen W Boateng GO. The enduring effects of early-childhood adversities and troubled sleep among Canadian adults: a population-based study. Sleep Med (2015) 16:760–7. 10.1016/j.sleep.2015.02.527 25953297

[48] Kajeepeta S Gelaye B Jackson CL Williams MA. Adverse childhood experiences are associated with adult sleep disorders: a systematic review. Sleep Med (2015) 16:320–30. 10.1016/j.sleep.2014.12.013 25777485 PMC4635027

[49] Chapman DP Liu Y Presley-Cantrell LR Edwards VJ Wheaton AG Perry GS Adverse childhood experiences and frequent insufficient sleep in 5 U.S. States, 2009: a retrospective cohort study. BMC Public Health (2013) 13:3. 10.1186/1471-2458-13-3 23286392 PMC3552999

[50] Agargun MY Kara H Ozer OA Selvi Y Kiran U Kiran S. Nightmares and dissociative experiences: the key role of childhood traumatic events. Psychiatry Clin Neurosci (2003) 57:139–45. 10.1046/j.1440-1819.2003.01093.x 12667159

[51] Agargun MY Sekeroglu MR Kara H Ozer OA Tombul T Kiran U Sleep-related violence and low serum cholesterol: a preliminary study. Psychiatry Clin Neurosci (2002) 56:195–8. 10.1046/j.1440-1819.2002.00954.x 11952924

[52] Cecil CA Viding E McCrory EJ Gregory AM. Distinct mechanisms underlie associations between forms of childhood maltreatment and disruptive nocturnal behaviors. Dev Neuropsychol (2015) 40:181–99. 10.1080/87565641.2014.983636 26151615

[53] Tomasdottir MO Sigurdsson JA Petursson H Kirkengen AL Krokstad S McEwen B Self reported childhood difficulties, adult multimorbidity and allostatic load. A cross-sectional analysis of the Norwegian HUNT Study. PLoS One (2015) 10:e0130591. 10.1371/journal.pone.0130591 26086816 PMC4472345

[54] Fisher HL Lereya ST Thompson A Lewis G Zammit S Wolke D. Childhood parasomnias and psychotic experiences at age 12 years in a United Kingdom birth cohort. Sleep (2014) 37:475–82. 10.5665/sleep.3478 24587569 PMC3920312

[55] Kales A Soldatos CR Caldwell AB Charney DS Kales JD Markel D Nightmares: clinical characteristics and personality patterns. Am J Psychiatry (1980) 137:1197–201. 10.1176/ajp.137.10.1197 7416265

[56] Duval M McDuff P Zadra A. Nightmare frequency, nightmare distress, and psychopathology in female victims of childhood maltreatment. J Nerv Ment Dis (2013) 201:767–72. 10.1097/NMD.0b013e3182a214a1 23995032

[57] Cuddy MA Belicki K Nightmare frequency and related sleep disturbance as indicators of a history of sexual abuse. Dreaming (1992) 2:15–22. 10.1037/h0094344

[58] Chambers E Belicki K. Using sleep dysfunction to explore the nature of resilience in adult survivors of childhood abuse or trauma. Child Abuse Negl (1998) 22:753–8. 10.1016/S0145-2134(98)00059-3 9717612

[59] Punamäki R-L Ali KJ Ismahil KH Nuutinen J Trauma, dreaming, and psychological distress among Kurdish children. Dreaming (2005) 15:178–94. 10.1037/1053-0797.15.3.178

[60] Lereya ST Winsper C Tang NK Wolke D Sleep problems in childhood and borderline personality disorder symptoms in early adolescence. J Abnormal Child Psychol (2017) 45(1):193–206. 10.1007/s10802-016-0158-4 PMC5219009 27108717

[61] Simard V Nielsen TA Tremblay RE Boivin M Montplaisir JY Longitudinal study of bad dreams in preschool children: prevalence, demographic correlates, risk and protective factors. Sleep (2008) 31:62–70. 10.1093/sleep/31.1.62 18220079 PMC2225564

[62] Csoka S Simor P Szabo G Kopp MS Bodizs R. Early maternal separation, nightmares, and bad dreams: results from the Hungarostudy Epidemiological Panel. Attach Human Dev (2011) 13:125–40. 10.1080/14616734.2011.553991 21390906

[63] Nielsen T Paquette T Carr M Sainte-Onge K Marquis LP Blanchette-Carriere C Prior trauma and adversity predict idiopathic nightmares even in healthy controls. Sleep (2016) 39(Suppl):A226.

[64] van der Kolk BA Smyth N Trauma Assessment Packet [CD-ROM]. Buffalo, NY: University of Buffalo School of Social Work: The Trauma Center at Justice Resource Institute (2010).

[65] Steine IM Krystal JH Nordhus IH Bjorvatn B Harvey AG Eid J Insomnia, nightmare frequency, and nightmare distress in victims of sexual abuse: the role of perceived social support and abuse characteristics. J Interpers Violence (2012) 27:1827–43. 10.1177/0886260511430385 22204947

[66] Hartmann E, editor. The biology of the nightmare. The Nightmare: The Psychology and Biology of Terrifying Dreams. New York: Basic Books (1984). p. 246–72.

[67] Hartmann E, editor. Post-traumatic nightmares. The Nightmare: The Psychology and Biology of Terrifying Dreams. New York: Basic Books (1984). p. 185–219.

[68] McCann SJ Stewin LL Short RH Frightening dream frequency and birth order. Individ Psychol (1990) 46:304–10.

[69] McCann SJH Stewin LL Frightening dreams and birth order. Individ Psychol (1987) 43:56–8.

[70] Brink T Matlock FE Nightmares and birth order: an empirical study. Individ Psychol (1982) 38:47–9.

[71] Eckstein D Aycock KJ Sperber MA McDonald J Van Wiesner V III Watts RE A review of 200 birth-order studies: lifestyle characteristics. J Individ Psychol (2010) 66:408–34.

[72] Hartmann E, editor. Dreams and Nightmares. New York: Plenum (1998).

[73] Gauchat A Seguin JR Zadra A. Prevalence and correlates of disturbed dreaming in children. Pathol Biol (Paris) (2014) 62:311–8. 10.1016/j.patbio.2014.05.016 25108315

[74] Schredl M Fricke-Oerkermann L Mitschke A Wiater A Lehmkuhl G. Longitudinal study of nightmares in children: stability and effect of emotional symptoms. Child Psychiatry Hum Dev (2009) 40:439–49. 10.1007/s10578-009-0136-y 19280336

[75] Mota N Tsai J Kirwin PD Harpaz-Rotem I Krystal JH Southwick SM Late-life exacerbation of PTSD symptoms in US veterans: results from the National Health and Resilience in Veterans Study. J Clin Psychiatry (2016) 77:348–54. 10.4088/JCP.15m10101 27046308

[76] Bielas H Barra S Skrivanek C Aebi M Steinhausen HC Bessler C The associations of cumulative adverse childhood experiences and irritability with mental disorders in detained male adolescent offenders. Child Adolesc Psychiatry Ment Health (2016) 10:34. 10.1186/s13034-016-0122-7 27688799 PMC5034668

[77] Sledjeski EM Speisman B Dierker LC. Does number of lifetime traumas explain the relationship between PTSD and chronic medical conditions? Answers from the National Comorbidity Survey-Replication (NCS-R). J Behav Med (2008) 31:341–9. 10.1007/s10865-008-9158-3 18553129 PMC2659854

[78] Callaghan BL Tottenham N The stress acceleration hypothesis: effects of early-life adversity on emotion circuits and behavior. Curr Opin Behav Sci (2016) 7:76–81. 10.1016/j.cobeha.2015.11.018 PMC5890821 29644262

[79] Fox MW Integrative Development of Brain and Behavior in the Dog. Oxford, England, UK: Chicago Press (1971).

[80] Rubin DC. The distribution of early childhood memories. Memory (2000) 8:265–9. 10.1080/096582100406810 10932795

[81] Freud S Three essays on the theory of sexuality. In: Strachey TJ, editor. The Standard Edition of the Complete Psychological Works of Sigmund Freud (Vol. 7). New York, NY: The Hogarth Press (1920). p. 125–245.

[82] Ellis BJ. Timing of pubertal maturation in girls: an integrated life history approach. Psychol Bull (2004) 130:920–58. 10.1037/0033-2909.130.6.920 15535743

[83] Cowan CS Callaghan BL Richardson R. Acute early-life stress results in premature emergence of adult-like fear retention and extinction relapse in infant rats. Behav Neurosci (2013) 127:703–11. 10.1037/a0034118 24128359

[84] Gee DG Gabard-Durnam L Telzer EH Humphreys KL Goff B Shapiro M Maternal buffering of human amygdala-prefrontal circuitry during childhood but not during adolescence. Psychol Sci (2014) 25:2067–78. 10.1177/0956797614550878 25280904 PMC4377225

[85] Richardson R Cowan CSM Callaghan BL Kan JM Effects of early-life stress on fear memory in the developing rat. Curr Opin Behav Sci (2016) 7:15–20. 10.1016/j.cobeha.2015.10.003

[86] Tottenham N. Social scaffolding of human amygdala-mPFC circuit development. Soc Neurosci (2015) 10:489–99. 10.1080/17470919.2015.1087424 26313424 PMC4890612

[87] Belsky J Steinberg L Draper P. Childhood experience, interpersonal development, and reproductive strategy: and evolutionary theory of socialization. Child Dev (1991) 62:647–70. 10.2307/1131166 1935336

[88] Del Giudice M Ellis BJ Shirtcliff EA. The Adaptive Calibration Model of stress responsivity. Neurosci Biobehav Rev (2011) 35:1562–92. 10.1016/j.neubiorev.2010.11.007 21145350 PMC3068241

[89] McLaughlin KA Green JG Gruber MJ Sampson NA Zaslavsky AM Kessler RC. Childhood adversities and adult psychopathology in the National Comorbidity Survey Replication (NCS-R) III: associations with functional impairment related to DSM-IV disorders. Psychol Med (2010) 40:847–59. 10.1017/S0033291709991115 19732483 PMC2847368

[90] Callaghan BL Sullivan RM Howell B Tottenham N The international society for developmental psychobiology Sackler symposium: early adversity and the maturation of emotion circuits – a cross-species analysis. Dev Psychobiol (2014) 56:1635–50. 10.1002/dev.21260 25290865 PMC4831705

[91] Callaghan BL Li S Richardson R The elusive engram: what can infantile amnesia tell us about memory? Trends Neurosci (2014) 37:47–53. 10.1016/j.tins.2013.10.007 24287309

[92] Gee DG Casey BJ. The impact of developmental timing for stress and recovery. Neurobiol Stress (2015) 1:184–94. 10.1016/j.ynstr.2015.02.001 25798454 PMC4363736

[93] Henri V Henri C On our earliest recollections of childhood. Psychol Rev (1895) 2:215–6.

[94] Freud S In: Strachey J, editor. The Psychopathology of Everyday Life (Vol. 5). Harmondsworth, Middlesex, England: Penguin (1938).

[95] Freud S Breuer J On the Psychical Mechanism of Hysterical Phenomena: Preliminary Communication, Standard Edition, (Vol. 2). London, England: Hogarth Press (1893).

[96] Ganella DE Kim JH Developmental rodent models of fear and anxiety: from neurobiology to pharmacology. Br J Pharmacol (2014) 171:4556–74. 10.1111/bph.12643 24527726 PMC4209932

[97] Jones E On the Nightmare. New York: Grove Press (1910).

[98] Bowlby J Attachment and Loss. Volume III. Loss, Sadness and Depression. New York, NY: Basic Books (1980).

[99] Werner H Kaplan B Symbol Formation. Oxford, England: Wiley (1963).

[100] Campbell BA Spear NE Ontogeny of memory. Psychol Rev (1972) 79:215–36. 10.1037/h0032690 4341445

[101] Dudycha GJ Dudycha MM Childhood memories: a review of the literature. Psychol Bull (1941) 38:668–82. 10.1037/h0055678

[102] Campbell BA Campbell EH Retention and extinction of learned fear in infant and adult rats. J Comp Physiol Psychol (1962) 55:1–8. 10.1037/h0049182 13876002

[103] Bachevalier J Mishkin M. An early and a late developing system for learning and retention in infant monkeys. Behav Neurosci (1984) 98:770–8. 10.1037/0735-7044.98.5.770 6541498

[104] Howe ML, editor. Infantile amnesia in human and nonhuman animals. The Nature of Early Memory: An Adaptive Theory of the Genesis and Development of Memory. New York, NY: Oxford University Press (2011). p. 47–66.

[105] Scarf D Gross J Colombo M Hayne H. To have and to hold: episodic memory in 3- and 4-year-old children. Dev Psychobiol (2013) 55:125–32. 10.1002/dev.21004 22213009

[106] Costello EJ Erkanli A Fairbank JA Angold A. The prevalence of potentially traumatic events in childhood and adolescence. J Trauma Stress (2002) 15:99–112. 10.1023/A:1014851823163 12013070

[107] Frans O Rimmo PA Aberg L Fredrikson M Trauma exposure and post-traumatic stress disorder in the general population. Acta Psychiatr Scand (2005) 111:291–9. 10.1111/j.1600-0447.2004.00463.x 15740465

[108] Terr L What happens to early memories of trauma? A study of twenty children under age five at the time of documented traumatic events. J Am Acad Child Adolesc Psychiatry (1988) 27:96–104. 10.1097/00004583-198801000-00015 3343214

[109] Howe ML Courage ML Peterson C How can I remember when “I” wasn’t there: long-term retention of traumatic experiences and emergence of the cognitive self. Conscious Cogn (1994) 3:327–55. 10.1006/ccog.1994.1019

[110] Peterson C Rideout R. Memory for medical emergencies experienced by 1- and 2-year-olds. Dev Psychol (1998) 34:1059–72. 10.1037/0012-1649.34.5.1059 9779751

[111] Cordón IM Pipe M-E Sayfan L Melinder A Goodman GS Memory for traumatic experiences in early childhood. Dev Rev (2004) 24:101–32. 10.1016/j.dr.2003.09.003

[112] Foulkes D. Home and laboratory dreams: four empirical studies and a conceptual reevaluation. Sleep (1979) 2:233–51. 232567

[113] Foulkes D Children’s Dreams: Longitudinal Studies. New York, NY: John Wiley & Sons (1982).

[114] Foulkes D Children’s Dreaming and the Development of Consciousness. Cambridge: Harvard University Press (1999).

[115] Terr LC Nightmares in children. In: Guilleminault C, editor. Sleep and Its Disorders in Children. New York: Raven Press (1987). p. 231–42.

[116] Mack JE Nightmares, conflict, and ego development in childhood. Int J Psycho Anal (1965) 46:403–28. 5866078

[117] Petit D Pennestri MH Paquet J Desautels A Zadra A Vitaro F Childhood sleepwalking and sleep terrors: a longitudinal study of prevalence and familial aggregation. JAMA Pediatr (2015) 169:653–8. 10.1001/jamapediatrics.2015.127 25938617

[118] Terr LC. Childhood traumas: an outline and overview. Am J Psychiatry (1991) 148:10–20. 10.1176/ajp.148.1.10 1824611

[119] Zadra A Desautels A Petit D Montplaisir J. Somnambulism: clinical aspects and pathophysiological hypotheses. Lancet Neurol (2013) 12:285–94. 10.1016/S1474-4422(12)70322-8 23415568

[120] Joncas S Zadra A Paquet J Montplaisir J. The value of sleep deprivation as a diagnostic tool in adult sleepwalkers. Neurology (2002) 58:936–40. 10.1212/WNL.58.6.936 11914411

[121] Denton DA McKinley MJ Farrell M Egan GF. The role of primordial emotions in the evolutionary origin of consciousness. Conscious Cogn (2009) 18:500–14. 10.1016/j.concog.2008.06.009 18701321

[122] Izard CE Woodburn EM Finlon KJ Extending emotion science to the study of discrete emotions in infants. Emot Rev (2010) 2:134–6. 10.1177/1754073909355003

[123] Izard CE. Emotion theory and research: highlights, unanswered questions, and emerging issues. Annu Rev Psychol (2009) 60:1–25. 10.1146/annurev.psych.60.110707.163539 18729725 PMC2723854

[124] Izard CE Fantauzzo CA Castle JM Haynes OM Rayias MF Putnam PH The ontogeny and significance of infants’ facial expressions in the first 9 months of life. Dev Psychol (1995) 31:997–1013. 10.1037/0012-1649.31.6.997

[125] Zadra A Pilon M Donderi DC. Variety and intensity of emotions in nightmares and bad dreams. J Nerv Ment Dis (2006) 194:249–54. 10.1097/01.nmd.0000207359.46223.dc 16614545

[126] Freud S The Interpretation of Dreams. New York: Basic Books (1900).

[127] Bauer PJ Doydum AO Pathman T Larkina M Guler OE Burch M It’s all about location, location, location: children’s memory for the “where” of personally experienced events. J Exp Child Psychol (2012) 113:510–22. 10.1016/j.jecp.2012.06.007 23010356 PMC3478447

[128] Glenberg AM Hayes J. Contribution of embodiment to solving the riddle of infantile amnesia. Front Psychol (2016) 7:10. 10.3389/fpsyg.2016.00010 26834683 PMC4724724

[129] Terr LC Childhood traumas: an outline and overview. Focus (2003) 1:322–33. 10.1176/foc.1.3.322 1824611

[130] Haroutunian V Riccio DC. Reduction of ontogenetic retention decrements in rats by pretraining stressful experiences. J Comp Physiol Psychol (1979) 93:501–11. 10.1037/h0077567 479395

[131] Nielsen T When was your earliest dream? Association of very early dream recall with frequent current nightmares supports a stress-acceleration explanation of nightmares. Dreaming (2017). 10.1037/drm0000051

[132] Samuel N Jacob R Eilon Y Mashiach T Shavit I. Falls in young children with minor head injury: a prospective analysis of injury mechanisms. Brain Inj (2015) 29:946–50. 10.3109/02699052.2015.1017005 25955119

[133] Agran PF Anderson C Winn D Trent R Walton-Haynes L Thayer S. Rates of pediatric injuries by 3-month intervals for children 0 to 3 years of age. Pediatrics (2003) 111:e683–92. 10.1542/peds.111.6.e683 12777586

[134] Robert G Zadra A. Thematic and content analysis of idiopathic nightmares and bad dreams. Sleep (2014) 37:409–17. 10.5665/sleep.3426 24497669 PMC3900621

[135] Griffith RM Miyagi O Tago A The universality of typical dreams: Japanese vs. Americans. Am Anthropologist (1958) 60:1173–9. 10.1525/aa.1958.60.6.02a00110

[136] Nielsen TA Zadra AL Simard V Saucier S Stenstrom P Smith C The typical dreams of Canadian University Students. Dreaming (2003) 13:211–35. 10.1023/B:DREM.0000003144.40929.0b

[137] Schredl M Ciric P Gotz S Wittmann L. Typical dreams: stability and gender differences. J Psychol (2004) 138:485–94. 10.3200/JRLP.138.6.485-494 15612605

[138] Yu CK-C Typical dreams experienced by Chinese people. Dreaming (2008) 18:1–10. 10.1037/1053-0797.18.1.1

[139] Zadra AL Recurrent dreams: their relation to life events. In: Barrett D, editor. Trauma and Dreams. Cambridge, MA: Harvard University Press (1996). p. 231–47.

[140] Frankenhuis WE House B Barrett HC Johnson SP. Infants’ perception of chasing. Cognition (2013) 126:224–33. 10.1016/j.cognition.2012.10.001 23121710 PMC3529835

[141] Carr M Blanchette-Carriere C Solomonova E Paquette T Nielsen T Intensified daydreams and nap dreams in frequent nightmare sufferers. Dreaming (2016) 26:119–31. 10.1037/drm0000024

[142] Carr M Blanchette-Carriere C Marquis LP Ting CT Nielsen T. Nightmare sufferers show atypical emotional semantic associations and prolonged REM sleep-dependent emotional priming. Sleep Med (2016) 20:80–7. 10.1016/j.sleep.2015.11.013 27318230

[143] Li S Callaghan BL Richardson R. Infantile amnesia: forgotten but not gone. Learn Mem (2014) 21:135–9. 10.1101/lm.031096.113 24532837 PMC3929851

[144] Li S Richardson R. Traces of memory: reacquisition of fear following forgetting is NMDAr-independent. Learn Mem (2013) 20:174–82. 10.1101/lm.029504.112 23504515

[145] Kandel ER. The molecular biology of memory storage: a dialogue between genes and synapses. Science (2001) 294:1030–8. 10.1126/science.1067020 11691980

[146] Wiltgen BJ Wood AN Levy B. The cellular mechanisms of memory are modified by experience. Learn Mem (2011) 18:747–50. 10.1101/lm.024026.111 22086392

[147] Poulos AM Reger M Mehta N Zhuravka I Sterlace SS Gannam C Amnesia for early life stress does not preclude the adult development of posttraumatic stress disorder symptoms in rats. Biol Psychiatry (2014) 76:306–14. 10.1016/j.biopsych.2013.10.007 24231200 PMC3984614

[148] Krikorian R Layton BS. Implicit memory in posttraumatic stress disorder with amnesia for the traumatic event. J Neuropsychiatry Clin Neurosci (1998) 10:359–62. 10.1176/jnp.10.3.359 9706546

[149] Layton BS Krikorian R Dori G Martin GA Wardi K. Posttraumatic stress disorder with amnesia following asphyxiation. Ann N Y Acad Sci (2006) 1071:488–90. 10.1196/annals.1364.048 16891604

[150] Kim JH Li S Hamlin AS McNally GP Richardson R Phosphorylation of mitogen-activated protein kinase in the medial prefrontal cortex and the amygdala following memory retrieval or forgetting in developing rats. Neurobiol Learn Mem (2012) 97:59–68. 10.1016/j.nlm.2011.09.005 21963362

[151] Slopen N Kubzansky LD McLaughlin KA Koenen KC. Childhood adversity and inflammatory processes in youth: a prospective study. Psychoneuroendocrinology (2013) 38:188–200. 10.1016/j.psyneuen.2012.05.013 22727478 PMC3632283

[152] Slopen N McLaughlin KA Dunn EC Koenen KC Childhood adversity and cell-mediated immunity in young adulthood: does type and timing matter? Brain Behav Immun (2013) 28:63–71. 10.1016/j.bbi.2012.10.018 23108062 PMC4180230

[153] Callaghan BL Richardson R. The effect of adverse rearing environments on persistent memories in young rats: removing the brakes on infant fear memories. Transl Psychiatry (2012) 2:e138. 10.1038/tp.2012.65 22781171 PMC3410617

[154] McLaughlin KA Greif Green J Gruber MJ Sampson NA Zaslavsky AM Kessler RC. Childhood adversities and first onset of psychiatric disorders in a national sample of US adolescents. Arch Gen Psychiatry (2012) 69:1151–60. 10.1001/archgenpsychiatry.2011.2277 23117636 PMC3490224

[155] Kim JH Richardson R. New findings on extinction of conditioned fear early in development: theoretical and clinical implications. Biol Psychiatry (2010) 67:297–303. 10.1016/j.biopsych.2009.09.003 19846065

[156] Bjorklund DF The role of immaturity in human development. Psychol Bull (1997) 122:153–69. 10.1037/0033-2909.122.2.153 9283298

[157] Clawson BC Durkin J Aton SJ. Form and function of sleep spindles across the lifespan. Neural Plast (2016) 2016:6936381. 10.1155/2016/6936381 27190654 PMC4848449

[158] Lindemann C Ahlbeck J Bitzenhofer SH Hanganu-Opatz IL. Spindle activity orchestrates plasticity during development and sleep. Neural Plast (2016) 2016:5787423. 10.1155/2016/5787423 27293903 PMC4884844

[159] Diekelmann S Born J. The memory function of sleep. Nat Rev Neurosci (2010) 11:114–26. 10.1038/nrn2762 20046194

[160] Ulrich D. Sleep spindles as facilitators of memory formation and learning. Neural Plast (2016) 2016:1796715. 10.1155/2016/1796715 27119026 PMC4826925

[161] Laventure S Fogel S Lungu O Albouy G Sevigny-Dupont P Vien C NREM2 and sleep spindles are instrumental to the consolidation of motor sequence memories. PLoS Biol (2016) 14:e1002429. 10.1371/journal.pbio.1002429 27032084 PMC4816304

[162] Kurdziel L Duclos K Spencer RM. Sleep spindles in midday naps enhance learning in preschool children. Proc Natl Acad Sci U S A (2013) 110:17267–72. 10.1073/pnas.1306418110 24062429 PMC3808582

[163] Kaestner EJ Wixted JT Mednick SC. Pharmacologically increasing sleep spindles enhances recognition for negative and high-arousal memories. J Cogn Neurosci (2013) 25:1597–610. 10.1162/jocn_a_00433 23767926

[164] Mikoteit T Brand S Beck J Perren S Von Wyl A Von Klitzing K Visually detected NREM Stage 2 sleep spindles in kindergarten children are associated with current and future emotional and behavioural characteristics. J Sleep Res (2012) 22:129–36. 10.1111/j.1365-2869.2012.01058.x 23046065

[165] Mikoteit T Brand S Beck J Perren S von Wyl A von Klitzing K Visually detected NREM Stage 2 sleep spindles in kindergarten children are associated with stress challenge and coping strategies. World J Biol Psychiatry (2012) 13:259–68. 10.3109/15622975.2011.562241 21486109

[166] Nielsen T Carr M Blanchette-Carrière C Marquis LP Dumel G Solomonova E NREM sleep spindles are associated with dream recall. Sleep Spindles Cortical Up States (2016) 1:1–15. 10.1556/2053.1.2016.003

[167] Carr M Picard-Deland C Paquette T Blanchette-Carriere C Sainte-Onge K Nielsen T Altered NREM spindle features in nightmare sufferers. 34th Annual Conference of the International Association for the Study of Dreams. Anaheim, CA (2017).

[168] Scholle S Zwacka G Scholle HC. Sleep spindle evolution from infancy to adolescence. Neurophysiol Clin (2007) 118:1525–31. 10.1016/j.clinph.2007.03.007 17475551

[169] Lenard HG Pennigstorff H Alterations in the sleep patterns of infants and young children following acute head injuries. Acta Paediatr Scand (1970) 59:565–71. 10.1111/j.1651-2227.1970.tb16809.x 4318408

[170] Roffwarg HP Dement WC Fisher C Preliminary observations of the sleep dream pattern in neonates, infants, children and adults. Monogr Child Psychiatry (1964) 2:60–72.

[171] McClain IJ Lustenberger C Achermann P Lassonde JM Kurth S LeBourgeois MK. Developmental changes in sleep spindle characteristics and sigma power across early childhood. Neural Plast (2016) 2016:3670951. 10.1155/2016/3670951 27110405 PMC4826705

[172] McNamara P Dowdall J Auerbach S. Rem sleep, early experience, and the development of reproductive strategies. Hum Nat (2002) 13:405–35. 10.1007/s12110-002-1001-x 26193088

[173] Reite M Short RA. Nocturnal sleep in separated monkey infants. Arch Gen Psychiatry (1978) 35:1247–53. 10.1001/archpsyc.1978.01770340097011 211985

[174] Dugovic C Maccari S Weibel L Turek FW Van Reeth O. High corticosterone levels in prenatally stressed rats predict persistent paradoxical sleep alterations. J Neurosci (1999) 19:8656–64. 10493766 10.1523/JNEUROSCI.19-19-08656.1999 PMC6783036

[175] Sampath D Sabitha KR Hegde P Jayakrishnan HR Kutty BM Chattarji S A study on fear memory retrieval and REM sleep in maternal separation and isolation stressed rats. Behav Brain Res (2014) 273:144–54. 10.1016/j.bbr.2014.07.034 25084041

[176] Rao U McGinty DJ Shinde A McCracken JT Poland RE. Prenatal stress is associated with depression-related electroencephalographic sleep changes in adult male rats: a preliminary report. Prog Neuro Psychopharmacol Biol Psychiatry (1999) 23:929–39. 10.1016/S0278-5846(99)00036-6 10509385

[177] Tiba PA Tufik S Suchecki D. Long lasting alteration in REM sleep of female rats submitted to long maternal separation. Physiol Behav (2008) 93:444–52. 10.1016/j.physbeh.2007.10.001 17997461

[178] Mrdalj J Pallesen S Milde AM Jellestad FK Murison R Ursin R Early and later life stress alter brain activity and sleep in rats. PLoS One (2013) 8:e69923. 10.1371/journal.pone.0069923 23922857 PMC3724678

[179] Heitkemper MM Cain KC Burr RL Jun SE Jarrett ME. Is childhood abuse or neglect associated with symptom reports and physiological measures in women with irritable bowel syndrome? Biol Res Nurs (2011) 13:399–408. 10.1177/1099800410393274 21196423 PMC3569490

[180] McNamara P Pace-Schott EF Johnson P Harris E Auerbach S. Sleep architecture and sleep-related mentation in securely and insecurely attached people. Attach Hum Dev (2011) 13:141–54. 10.1080/14616734.2011.553999 21390907 PMC3060050

[181] Fonzo GA Ramsawh HJ Flagan TM Simmons AN Sullivan SG Allard CB Early life stress and the anxious brain: evidence for a neural mechanism linking childhood emotional maltreatment to anxiety in adulthood. Psychol Med (2016) 46:1037–54. 10.1017/S0033291715002603 26670947 PMC4795156

[182] Tyrka AR Parade SH Price LH Kao HT Porton B Philip NS Alterations of mitochondrial DNA copy number and telomere length with early adversity and psychopathology. Biol Psychiatry (2016) 79:78–86. 10.1016/j.biopsych.2014.12.025 25749099 PMC4503518

[183] French JA Carp SB. Early-life social adversity and developmental processes in nonhuman primates. Curr Opin Behav Sci (2016) 7:40–6. 10.1016/j.cobeha.2015.11.004 26858971 PMC4742359

[184] Farrell MR Holland FH Shansky RM Brenhouse HC. Sex-specific effects of early life stress on social interaction and prefrontal cortex dendritic morphology in young rats. Behav Brain Res (2016) 310:119–25. 10.1016/j.bbr.2016.05.009 27180166 PMC12878820

[185] Callaghan BL Richardson R. Early experiences and the development of emotional learning systems in rats. Biol Mood Anxiety Disord (2013) 3:8. 10.1186/2045-5380-3-8 23575272 PMC3637360

[186] Ehrlich DE Josselyn SA. Plasticity-related genes in brain development and amygdala-dependent learning. Genes Brain Behav (2016) 15:125–43. 10.1111/gbb.12255 26419764

[187] Hartley CA Lee FS. Sensitive periods in affective development: nonlinear maturation of fear learning. Neuropsychopharmacology (2015) 40:50–60. 10.1038/npp.2014.179 25035083 PMC4262897

[188] Marstaller L Burianova H Reutens DC. Adaptive contextualization: a new role for the default mode network in affective learning. Hum Brain Mapp (2017) 38(2):1082–91. 10.1002/hbm.23442 27767246 PMC6867087

[189] Chow HM Horovitz SG Carr WS Picchioni D Coddington N Fukunaga M Rhythmic alternating patterns of brain activity distinguish rapid eye movement sleep from other states of consciousness. Proc Natl Acad Sci U S A (2013) 110:10300–5. 10.1073/pnas.1217691110 23733938 PMC3690889

[190] Domhoff GW Fox KC. Dreaming and the default network: a review, synthesis, and counterintuitive research proposal. Conscious Cogn (2015) 33:342–53. 10.1016/j.concog.2015.01.019 25723600

[191] Fox KC Nijeboer S Solomonova E Domhoff GW Christoff K. Dreaming as mind wandering: evidence from functional neuroimaging and first-person content reports. Front Hum Neurosci (2013) 7:412. 10.3389/fnhum.2013.00412 23908622 PMC3726865

[192] Pace-Schott E The frontal lobes and dreaming. In: Barrett D McNamara P, editors. The New Science of Dreaming: Content, Recall and Personality Correlates (Vol. 1). Westport, CT: Praeger (2007). p. 115–54.

[193] Fullana MA Harrison BJ Soriano-Mas C Vervliet B Cardoner N Avila-Parcet A Neural signatures of human fear conditioning: an updated and extended meta-analysis of fMRI studies. Mol Psychiatry (2016) 21:500–8. 10.1038/mp.2015.88 26122585

[194] Izquierdo I Furini CR Myskiw JC. Fear memory. Physiol Rev (2016) 96:695–750. 10.1152/physrev.00018.2015 26983799

[195] Furini C Myskiw J Izquierdo I. The learning of fear extinction. Neurosci Biobehav Rev (2014) 47:670–83. 10.1016/j.neubiorev.2014.10.016 25452113

[196] Greco JA Liberzon I. Neuroimaging of fear-associated learning. Neuropsychopharmacology (2016) 41:320–34. 10.1038/npp.2015.255 26294108 PMC4677141

[197] Ahs F Kragel PA Zielinski DJ Brady R LaBar KS. Medial prefrontal pathways for the contextual regulation of extinguished fear in humans. Neuroimage (2015) 122:262–71. 10.1016/j.neuroimage.2015.07.051 26220745 PMC4618170

[198] Silvers JA Lumian DS Gabard-Durnam L Gee DG Goff B Fareri DS Previous institutionalization is followed by broader amygdala-hippocampal-PFC network connectivity during aversive learning in human development. J Neurosci (2016) 36:6420–30. 10.1523/JNEUROSCI.0038-16.2016 27307231 PMC5015779

[199] Carhart-Harris RL Leech R Williams TM Erritzoe D Abbasi N Bargiotas T Implications for psychedelic-assisted psychotherapy: functional magnetic resonance imaging study with psilocybin. Br J Psychiatry (2012) 200:238–44. 10.1192/bjp.bp.111.103309 22282432

[200] Cheetham M Hanggi J Jancke L. Identifying with fictive characters: structural brain correlates of the personality trait ‘fantasy’. Soc Cogn Affect Neurosci (2014) 9:1836–44. 10.1093/scan/nst179 24464847 PMC4221223

[201] Fuentemilla L Barnes GR Duzel E Levine B. Theta oscillations orchestrate medial temporal lobe and neocortex in remembering autobiographical memories. Neuroimage (2014) 85(Pt 2):730–7. 10.1016/j.neuroimage.2013.08.029 23978597

[202] Huang Z Davis HI Yue Q Wiebking C Duncan NW Zhang J Increase in glutamate/glutamine concentration in the medial prefrontal cortex during mental imagery: a combined functional MRS and fMRI study. Hum Brain Mapp (2015) 36:3204–12. 10.1002/hbm.22841 26059006 PMC6869168

[203] Kleber B Birbaumer N Veit R Trevorrow T Lotze M. Overt and imagined singing of an Italian aria. Neuroimage (2007) 36:889–900. 10.1016/j.neuroimage.2007.02.053 17478107

[204] D’Argembeau A Jeunehomme O Majerus S Bastin C Salmon E. The neural basis of temporal order processing in past and future thought. J Cogn Neurosci (2015) 27:185–97. 10.1162/jocn_a_00680 24960045

[205] Lenggenhager B Halje P Blanke O. Alpha band oscillations correlate with illusory self-location induced by virtual reality. Eur J Neurosci (2011) 33:1935–43. 10.1111/j.1460-9568.2011.07647.x 21395867

